# Stable Singularity Formation for the Keller–Segel System in Three Dimensions

**DOI:** 10.1007/s00205-023-01947-9

**Published:** 2024-01-05

**Authors:** Irfan Glogić, Birgit Schörkhuber

**Affiliations:** 1 Faculty of Mathematics, University of Vienna, Oskar-Morgenstern-Platz 1, 1090 Vienna, Austria; 2 Universität Innsbruck, Institut für Mathematik, Technikerstraße 13, 6020 Innsbruck, Austria

## Abstract

We consider the parabolic–elliptic Keller–Segel system in dimensions $d \geqq 3$, which is the mass supercritical case. This system is known to exhibit rich dynamical behavior including singularity formation via self-similar solutions. An explicit example was found more than two decades ago by Brenner et al. (Nonlinearity 12(4):1071–1098, 1999), and is conjectured to be nonlinearly radially stable. We prove this conjecture for $d=3$. Our approach consists of reformulating the problem in similarity variables and studying the Cauchy evolution in intersection Sobolev spaces via semigroup theory methods. To solve the underlying spectral problem, we use a technique we recently established in Glogić and Schörkhuber (Comm Part Differ Equ 45(8):887–912, 2020). To the best of our knowledge, this provides the first result on stable self-similar blowup for the Keller–Segel system. Furthermore, the extension of our result to any higher dimension is straightforward. We point out that our approach is general and robust, and can therefore be applied to a wide class of parabolic models.

## Introduction

We consider the system of equations

1.1 $$\begin{aligned} {\left\{ \begin{array}{ll} \partial _t u(t,x)= \Delta u(t,x) + \nabla \! \cdot \! (u(t,x) \nabla v(t,x)), \\ \Delta v(t,x) = u(t,x), \end{array}\right. } \end{aligned}$$

equipped with an initial condition $u(0,\cdot )=u_0$, for $u, v: [0,T) \times {\mathbb {R}}^d \rightarrow {\mathbb {R}}$ and some $T>0$. This model is frequently referred to as the parabolic–elliptic Keller–Segel system, named after the authors of [39], who introduced a system of coupled parabolic equations to describe chemotactic aggregation phenomena in biology. The parabolic–elliptic version (1.1) was derived later by Jäger and Luckhaus [37]. System (1.1) arises also as a simplified model for self-gravitating matter in stellar dynamics, with *u* representing the gas density and *v* the corresponding gravitational potential, see e.g. [1, 55].

The equation for *v* in (1.1) can be solved explicitly in terms of *u*, which reduces the system to a single (non-local) parabolic equation

1.2 $$\begin{aligned} \partial _t u(t,x)= \Delta u(t,x) + u(t,x)^2 + \nabla v_u(t,x) \nabla u(t,x), \end{aligned}$$

where $v_u = G *u$, with *G* denoting the fundamental solution of the Laplace equation. This equation is invariant under the scaling transformation $u \mapsto u_{\lambda }$,

$$\begin{aligned} u_{\lambda }(t,x):= \lambda ^{-2} u(t/\lambda ^2, x/\lambda ), \quad \lambda >0. \end{aligned}$$

Furthermore, assuming sufficient decay of *u* at infinity, the total mass

$$\begin{aligned} {\mathcal {M}}(u)(t) = \int _{{\mathbb {R}}^d} u(t,x) \textrm{d}x \end{aligned}$$

is conserved. Since ${\mathcal {M}}(u_{\lambda }) = \lambda ^{d-2} {\mathcal {M}}(u)(\cdot /\lambda ^2)$, the model is mass critical for $d=2$ and mass supercritical for $d \geqq 3$.

It is well known that Equation (1.1) admits finite-time blowup solutions in all space dimensions $d \geqq 2$, for which, in particular,

$$\begin{aligned} \lim _{t \rightarrow T^{-}} \Vert u(t,\cdot ) \Vert _{L^{\infty }({\mathbb {R}}^d)} = \infty \end{aligned}$$

for some $T >0$. This is natural in view of the phenomena that the model is supposed to describe, and there is a strong interest in understanding the structure of singularities. Consequently, there is a huge body of literature addressing this question for (1.1) and variants thereof, for a review see e.g. [35, 36].

Being the natural setting for biological applications, a lot of attention has centred around the mass critical case $d=2$. There, the $L^1$-norm of the stationary ground state solution *Q*, defined in (1.3) below, represents the threshold for singularity formation, see e.g. [7, 8, 17, 21]. Particular solutions that blow up in finite time via dynamical rescaling of *Q*,

1.3 $$\begin{aligned} u(t,x) \sim \frac{1}{\lambda (t)^2} Q\left( \frac{x}{\lambda (t)} \right) , \quad Q(x) = \frac{8}{(1+|x|^2)^2} \end{aligned}$$

with $\lambda (t) \rightarrow 0$ for $t \rightarrow T^-$, have been constructed for different blowup rates $\lambda$, see [13, 34, 48, 53]. In particular, in [13] it is shown that the blowup solution corresponding to

1.4 $$\begin{aligned} \lambda (t) = \kappa \sqrt{T-t} e^{-\sqrt{\frac{1}{2}| \log (T-t)|}} \end{aligned}$$

for a certain explicit constant $\kappa > 0$, is stable outside of radial symmetry. In addition to this, Mizoguchi [41] recently proved that for solutions with non-negative and radial initial data, (1.3)–(1.4) describes the universal blowup mechanism.

In comparison, the dynamics in the supercritical case $d \geqq 3$ are more complex; in particular, multiple blowup profiles are known to exist. In a recent work, Collot, Ghoul, Masmoudi and Nguyen [14] proved for all $d \geqq 3$ the existence of a blowup solution that concentrates in a thin layer outside the origin and implodes towards the center. Other known examples of singular behavior are provided by self-similar solutions, which are proven to exist in all dimensions $d \geqq 3$, see [9, 33, 51]. A particular example was found in closed form in [9], and is given by

1.5 $$\begin{aligned} u_T(t,x):=\frac{1}{T-t} U \left( \frac{x}{\sqrt{T-t}} \right) ~ \text { where } ~ U(x)=\frac{4(d-2)(2d+|x|^2)}{(2(d-2)+|x|^2)^2}.\nonumber \\ \end{aligned}$$

### The Main Result

To understand the role of the solution (1.5) for generic evolutions of (1.1), the authors of [9] performed numerical experiments and conjectured as a consequence that $u_T$ is nonlinearly radially stable. In spite of a number of results on the nature of blowup, this conjecture has remained open for more than two decades now. In the main result of this paper we prove this conjecture for $d=3$. More precisely, we show that there is an open set of radial initial data around $u_1(0,\cdot )=U$ for which the Cauchy evolution of (1.1) forms a singularity in finite time $T > 0$ by converging to $u_T$, i.e., to the profile *U* after self-similar rescaling. The formal statement is as follows:

#### Theorem 1.1

Let $d = 3$. There exists $\varepsilon >0$ such that, for any initial datum

1.6 $$\begin{aligned} u(0,\cdot )=U +\varphi _0, \end{aligned}$$

where $\varphi _0$ is a radial Schwartz function for which

$$\begin{aligned} \Vert \varphi _0 \Vert _{{H}^3({\mathbb {R}}^3)} < \varepsilon , \end{aligned}$$

there exists $T>0$ and a classical solution $u \in C^\infty ([0,T) \times {\mathbb {R}}^3)$ to (1.1), which blows up at the origin as $t \rightarrow T^{-}$. Furthermore, the following profile decomposition holds:

$$\begin{aligned} u(t,x)=\frac{1}{T-t}\left[ U\left( \frac{x}{\sqrt{T-t}}\right) + \varphi \left( t, \frac{x}{\sqrt{T-t}} \right) \right] \end{aligned}$$

where $$ \Vert \varphi (t,\cdot ) \Vert _{{H}^3({\mathbb {R}}^3)} \rightarrow 0 $$ as $$t \rightarrow T^-$$.

#### Remark 1.2

As will be apparent from the proof, the extension of this result to any higher dimension is straightforward. This involves developing the analogous well-posedness theory and solving the underlying spectral problem for a particular choice of $d \geqq 4$. We therefore restrict ourselves to the lowest dimension, and the physically most relevant case, $d=3$.

#### Remark 1.3

Due to the embedding $H^3({\mathbb {R}}^3) \hookrightarrow L^\infty ({\mathbb {R}}^3)$, the conclusion of the theorem implies that the evolution of the perturbation (1.6), when dynamically self-similarly rescaled, converges back to *U* in $L^\infty ({\mathbb {R}}^3)$. In other words,

$$\begin{aligned} (T-t)\, u (t,\sqrt{T-t}\, \cdot ) \rightarrow U \end{aligned}$$

uniformly on ${\mathbb {R}}^3$ as $t \rightarrow T^-$.

### Related Results for $d \geqq 3$

There are many works that treat the system (1.1) in higher dimensions. Here we give a short and noninclusive overview of some of the important developments.

Local existence and uniqueness of radial solutions for (1.1) holds in $L^{\infty }({\mathbb {R}}^d)$ as well as in other function spaces, see e.g. [3, 25]. Concerning global existence, various criteria are given in terms of critical (i.e. scaling invariant) norms. For example, it is known that initial data of small $L^{d/2}({\mathbb {R}}^d)$-norm lead to global (weak) solutions [15]. This result was later extended by Calvez, Corrias, and Ebde [10] to all data of norm less than a certain constant coming from the Gagliardo-Nierenberg inequality. For results in terms of the critical Morey norms, see e.g. [3, 4, 40]. Concerning the existence of finite time blowup, the aforementioned works [10, 15] give sufficient conditions in terms of the size of the second moment of the initial data. For an earlier result of that type see the work of Nagai [42]. For other, more recent results see [3, 5, 6, 43, 46, 52]. We point out, however, that in contrast to the $d=2$ case, for $d \geqq 3$ still no simple characterization of threshold for blowup in terms of a critical norm is known.

Concerning the structure of singularities, not much is known. It is straight– forward to conclude that blowup solutions of (1.1) satisfy $\liminf _{t \rightarrow T^{-}} \,(T-t) \Vert u(t,\cdot ) \Vert _{L^{\infty }({\mathbb {R}}^d)} >0$, see e.g. [44]. Accordingly, singular solutions are classified as *type I* if

$$\begin{aligned} \limsup _{t \rightarrow T^{-}} \,(T-t) \Vert u(t,\cdot ) \Vert _{L^{\infty }({\mathbb {R}}^d)} < \infty , \end{aligned}$$

and as *type II* otherwise. The first formal construction of type II blowup was performed by Herrero, Medina and Velázquez for $d=3$ in [32]; the singularity they construct consists of a smoothed-out shock wave concentrated in a ring that collapses into a Dirac mass the origin. This blowup mechanism was later observed numerically in [9] for higher dimensions as well, and is furthermore conjectured to be radially stable. A rigorous construction of this solution for all $d \geqq 3$ came only recently in the work of Collot, Ghoul, Masmoudi and Nguyen [14], who also prove its radial stability. In contrast to these results, if the initial profile of a blowup solution is radially non-increasing and of finite mass then the limiting spatial profile is very much unlike the Dirac mass, since it satisfies

$$\begin{aligned} C_1 |x|^{-2} \leqq \lim _{t \rightarrow T^-} u(t,x) \leqq C_2|x|^{-2} \end{aligned}$$

near the origin, as proven by Souplet and Winkler [52]. This is in particular the case for self-similar solutions, for which $\lim _{t \rightarrow T^-} u(t,x) = C|x|^{-2}$ at the blowup time. To add to the importance of self-similar solutions for understanding the structure of singularities, Giga, Mizoguchi and Senba [25] showed that any radial, non-negative type I blowup solution of (1.1) is asymptotically self-similar. For $3 \leqq d \leqq 9$, it is known that there are infinitely many similarity profiles, while for $d \geqq 10$ there is at least one, see [51]. However, a full classification of the set of self-similar (blowup) solutions, even in radial symmetry, is not available so far.

Finally, we note that in the two-dimensional case, any blowup solution is necessarily of type II, see e.g. [45]. In the mass subcritical case $d=1$ blowup has been excluded in [12].

### Outline of the Proof of the Main Result

Since we assume radial symmetry, i.e. $u(t,x) = {{\tilde{u}}}(t,|x|)$, we first reformulate (1.1) in terms of the reduced mass

$$\begin{aligned} {{\tilde{w}}}(t,r):=\frac{1}{2r^2} \int _{0}^{r} {{\tilde{u}}}(t,s)s^{2}\textrm{d}s. \end{aligned}$$

The advantage of this change of variable lies in the fact that it reduces (1.2) to a local semilinear heat equation on ${\mathbb {R}}^{5}$ for $w(t,x):= {{\tilde{w}}}(t,|x|)$

1.7 $$\begin{aligned} {\left\{ \begin{array}{ll} ~ \partial _t w - \Delta w = \Lambda w^2 + 6 w^2,\\ ~ w(0,\cdot )=w_0, \end{array}\right. } \end{aligned}$$

where $\Lambda f(x):=x \cdot \nabla f(x)$, and the initial datum is radial $w_0={\tilde{w}}(0,|\cdot |)$. Furthermore, similar to (1.2), Equation (1.7) obeys the scaling law

$$\begin{aligned} w(t,x) \mapsto w_{\lambda }(t,x):=\lambda ^{-2} w(t/\lambda ^2,x/\lambda ), \quad \lambda >0, \end{aligned}$$

which leaves the ${\dot{H}}^{\frac{1}{2}}({\mathbb {R}}^5)$ norm invariant. Additionally, the self-similar solution (1.5) turns into

$$\begin{aligned} w_T(t,x):=\frac{1}{T-t}\phi \left( \frac{x}{\sqrt{T-t}}\right) \quad \text {where} \quad \phi (x)=\frac{2}{2+|x|^2}. \end{aligned}$$

The bulk of our proof consists of showing stability of $w_T$. Then, by using the equivalence of norms of *u* and *w* we turn the obtained stability result into Theorem 1.1. In Sect. 2, as is customary in the study of self-similar solutions, we pass to similarity variables

$$\begin{aligned} \tau = \tau (t):= \log \left( \frac{T}{T-t}\right) , \quad \xi = \xi (t,x):=\frac{x}{\sqrt{T-t}}. \end{aligned}$$

We remark that the application of similarity variables in the study of blowup for nonlinear parabolic equations goes back to 1980’s and the early works of Giga and Kohn [22–24]. Note that the strip $[0,T)\times {\mathbb {R}}^5$ is mapped via $(t,x) \mapsto (\tau ,\xi )$ into the half-space $[0,\infty ) \times {\mathbb {R}}^5$. Furthermore, by rescaling the dependent variable $(T-t)w(t,x)=:\Psi (\tau ,\xi )$, the solution $w_T$ becomes $\tau$-independent, $\xi \mapsto \phi (\xi )$. Consequently, the problem of stability of finite time blowup via $w_T$ is turned into the problem of the asymptotic stability of the static solution $\phi$. To study evolutions near $\phi$, we consider the pertubation ansatz $\Psi (\tau ,\cdot ) = \phi + \psi (\tau )$, which yields the central evolution equation of this paper:

1.8 $$\begin{aligned} {\left\{ \begin{array}{ll} ~ \partial _\tau \psi (\tau ) = L \psi (\tau ) + N\big (\psi (\tau )\big ),\\ ~ \psi (0) = T w_0(\sqrt{T}\cdot )-\phi . \end{array}\right. } \end{aligned}$$

Here, the linear operator *L* and the remaining nonlinearity *N* are explicitly given as

1.9 $$\begin{aligned} L = \Delta - \frac{1}{2} \Lambda - 1 + 2\Lambda (\phi \, \cdot )+ 12 \phi \quad \text {and} \quad N(f)= \Lambda f^2 + 6 f^2. \end{aligned}$$

The next step is to establish a well-posedness theory for the Cauchy problem (1.8). To that end, in Sect. 3 we introduce the principal function space of the paper:

$$\begin{aligned} X^k:= \dot{H}^1_{\text {rad}}({\mathbb {R}}^5) \cap \dot{H}^k_{\text {rad}}({\mathbb {R}}^5), \quad k \geqq 3. \end{aligned}$$

To construct solutions to (1.8) in $X^k$, we take up the abstract semigroup approach. In Sect. 4 we concentrate on the linear version of (1.8). First, we prove that *L*, being initially defined on test functions, is closable, and its closure ${\mathcal {L}}$ generates a strongly continuous semigroup $S(\tau )$ on $X^k$. Our proof is based on the Lumer–Phillips theorem, and it involves a delicate construction of global, radial and decaying solutions to the Poisson type equation ${\mathcal {L}}f=g$. Thereby we establish existence of the linear flow near $\phi$. This flow exhibits growth in general, due to the existence of the unstable eigenvalue $\lambda =1$ of the generator ${\mathcal {L}}$. This instability is not a genuine one though, as it arises naturally, due to the time translation invariance of the problem. By combining the analysis of the linear evolution in $X^k$ with a thorough spectral analysis of ${\mathcal {L}}$ in a suitably weighted $L^2$-space, we prove the existence of a (non-orthogonal) rank-one projection ${\mathcal {P}}: X^k \rightarrow X^k$ relative to $\lambda =1$, such that $\ker {\mathcal {P}}$ in an invariant subspace for ${\mathcal {L}}$, and the linear evolution in $X^k$ decays exponentially on $\ker {\mathcal {P}}$. This is expressed formally in the central result of the linear theory, Theorem 4.1.

The existence of $S(\tau )$ allows us to express the nonlinear Equation (1.8) in the integral from

1.10 $$\begin{aligned} \psi (\tau ) = S(\tau )\psi (0) + \int _{0}^{\tau }S(\tau -s)N(\psi (s))\textrm{d}s. \end{aligned}$$

As is customary, we employ a fixed point argument to construct solutions to (1.10) in $X^k$. For this, we need a suitable Lipschitz continuity property of the nonlinear operator *N* in $X^k$. However, the space $X^k$ is not invariant under the action of *N*, due to the presence of the derivative nonlinearity, recall (1.9). Nevertheless, by exploiting the smoothing properties of $S(\tau )$, we show that the operator $f \mapsto S(\tau )N(f)$ is locally Lipschitz continuous in $X^k$, which will suffice for setting up a contraction scheme for (1.10). The proofs of the smoothing properties of $S(\tau )$ and those of the accompanying nonlinear Lipschitz estimates comprise the main content of Sect. 5.

With these technical results at hand, in Sect. 6 we use a fixed point argument to construct for (1.10) global, exponentially decaying strong $X^4$-solutions for small data. To deal with the growth stemming from the presence of ${\text {rg}}{\mathcal {P}}$ in the initial data, we employ a Lyapunov-Perron type argument, by means of which we also extract the blowup time *T*. In Sect. 7, we use regularity arguments to show that the constructed strong solutions are in fact classical. By translating the obtained result back to physical coordinates (*t*, *x*), we get stability of $w_T$. Finally, by means of the equivalence of norms of *w* and *u*, from this we derive Theorem 1.1.

We finalize with a remark that our methods can straightforwardly be generalized so as to treat (1.7) in any higher dimension $n \geqq 6$. This involves carrying out the analogous well-posedness theory for (1.8) in the high-dimensional counterpart of the space $X^k$

$$\begin{aligned} X_n^k:= {\dot{H}}_{\text {rad}}^{\left\lfloor \frac{n}{2}\right\rfloor -1}({\mathbb {R}}^n) \cap {\dot{H}}_{\text {rad}}^{k}({\mathbb {R}}^n), \quad k \geqq \left\lfloor \tfrac{n}{2}\right\rfloor + 1, \end{aligned}$$

along with using the same techniques to treat the underlying spectral problem.

### Notation and Conventions

We write ${\mathbb {N}}$ for the natural numbers $\{1,2,3, \dots \}$, ${\mathbb {N}}_0:= \{0\} \cup {\mathbb {N}}$. Furthermore, ${\mathbb {R}}^+:= \{x \in {\mathbb {R}}: x >0\}$. By $C_{c}^\infty ({\mathbb {R}}^d)$ we denote the space of smooth functions with compact support. In addition, we define $C_{\textrm{rad }}^{\infty }({\mathbb {R}}^d):= \{ u \in C^{\infty }({\mathbb {R}}^d): u \text { is radial}\}$ and analogously $C^{\infty }_{c,\textrm{rad }}({\mathbb {R}}^d)$. Also, ${\mathcal {S}}_{\text {rad}}({\mathbb {R}}^d)$ stands for the space of radial Schwartz functions. By $L^p(\Omega )$ for $\Omega \subseteq {\mathbb {R}}^d$, we denote the standard Lebesgue space. For the Fourier transform we use the following convention:

1.11 $$\begin{aligned} {\mathcal {F}}_d(f)(\xi ) = \frac{1}{(2 \pi )^{d/2}} \int _{{\mathbb {R}}^d} e^{- i \xi \cdot x} f(x) \textrm{d}x. \end{aligned}$$

For a closed linear operator $(L, {\mathcal {D}}(L))$, we denote by $\rho (L)$ the resolvent set, and for $\lambda \in \rho (L)$ we use the following convention for the resolvent operator $R_{L}(\lambda ):=(\lambda - L)^{-1}$. The spectrum is defined as $\sigma (L):= {\mathbb {C}}\setminus \rho (L)$. The notation $a\lesssim b$ means $a\leqq Cb$ for some $C>0$, and we write $a\simeq b$ if $a\lesssim b$ and $b \lesssim a$. We use the common notation $\langle x \rangle := \sqrt{1+|x|^2}$ also known as the *Japanese bracket*.

## Equation in Similarity Variables

To restrict to radial solutions of (1.1) we assume that $u(t,\cdot )={\tilde{u}}(t,|\cdot |)$ and $u_0={\tilde{u}}_0(|\cdot |)$. Then, we define the so-called reduced mass

$$\begin{aligned} {\tilde{w}}(t,r):=\frac{1}{2r^d} \int _{0}^{r}{{\tilde{u}}}(t,s)s^{d-1}\textrm{d}s, \end{aligned}$$

and let $n:=d+2$. The utility of the reduced mass is reflected in the fact that by means of the function $w(t,x):={\tilde{w}}(t,|x|)$, the initial value problem for the system (1.1) can be rewritten in the form of a single local semilinear heat equation in *w*

2.1 $$\begin{aligned} {\left\{ \begin{array}{ll} ~ \partial _t w - \Delta w = \Lambda w^2 + 2d w^2,\\ ~ w(0,\cdot )=w_0. \end{array}\right. } \end{aligned}$$

Here $$(t,x) \in [0,T) \times {\mathbb {R}}^{n}$$,

$$\begin{aligned} \Lambda f(x):=x \cdot \nabla f(x), \end{aligned}$$

and

2.2 $$\begin{aligned} w_0={{\tilde{w}}}_0(|\cdot |) \quad \text {for} \quad {\tilde{w}}_0(r)= \frac{1}{2r^d} \int _{0}^{r}{{\tilde{u}}}_0(s) s^{d-1}\textrm{d}s. \end{aligned}$$

Furthermore, the self-similar solution (1.5) turns into

$$\begin{aligned} w_T(t,x):=\frac{1}{T-t}\phi _n\left( \frac{x}{\sqrt{T-t}}\right) \quad \text {where} \quad \phi _n(x)=\frac{2}{2(n-4)+|x|^2}. \end{aligned}$$

### Similarity Variables

We pass to similarity variables

2.3 $$\begin{aligned} \tau = \tau (t):=\ln \left( \frac{T}{T-t} \right) , \quad \xi =\xi (t,x):=\frac{x}{\sqrt{T-t}}, \quad T>0. \end{aligned}$$

Note that transformation (2.3) maps the time slab $S_T:=[0,T) \times {\mathbb {R}}^n$ into the upper half-space $H_+:=[0,+\infty ) \times {\mathbb {R}}^n$. Also, we define the rescaled dependent variable

2.4 $$\begin{aligned} \Psi (\tau ,\xi ):=(T-t)w(t,x)=Te^{-\tau }w(T-Te^{-\tau },\sqrt{T}e^{-\frac{\tau }{2}}\xi ). \end{aligned}$$

Consequently, the evolution of *w* inside $S_T$ corresponds to the evolution of $\Psi$ inside $H_+$. Furthermore, since

2.5 $$\begin{aligned} \partial _t = \frac{e^\tau }{T}\left( \partial _{\tau } + \frac{1}{2}\Lambda \right) , \quad \Delta _x = \frac{e^\tau }{T}\Delta _\xi , \end{aligned}$$

we get that the nonlinear heat equation (2.1) transforms into

2.6 $$\begin{aligned} \left( \partial _\tau - \Delta + \frac{1}{2} \Lambda + 1 \right) \Psi = \Lambda \Psi ^2 + 2(n-2) \Psi ^2, \end{aligned}$$

with the initial datum

$$\begin{aligned} \Psi (0,\cdot )= Tw_0(\sqrt{T}\cdot ). \end{aligned}$$

For convenience, we denote

2.7 $$\begin{aligned} L_0:= \Delta - \frac{1}{2} \Lambda - 1. \end{aligned}$$

Now, we have that for all $n \geqq 5$ the function

$$\begin{aligned} \phi _n(\xi )= \frac{2}{2(n-4)+|\xi |^2} \end{aligned}$$

is a static solution to (2.6). To analyze stability properties of $\phi _n$, we study evolutions of initial data near $\phi _n$, and for that we consider the perturbation ansatz

$$\begin{aligned} \Psi (\tau ,\cdot ) = \phi _n + \psi (\tau ). \end{aligned}$$

This leads to the central evolution equation of the paper,

2.8 $$\begin{aligned} \partial _\tau \psi (\tau ) = \big ( L_0 + L' \big )\psi (\tau ) + N\big (\psi (\tau )\big ), \end{aligned}$$

where

2.9 $$\begin{aligned} L'f = 2\Lambda (\phi _n f)+4 (n-2) \phi _n f \quad \text {and} \quad N(f)= \Lambda f^2 + 2(n-2) f^2. \end{aligned}$$

Furthermore, we write the initial datum as

2.10 $$\begin{aligned} \psi (0)= \Psi (0,\cdot ) - \phi _n = \big (T w_0(\sqrt{T}\cdot )-w_0 \big ) + v =: U(v,T), \end{aligned}$$

where, for convenience, we denote

2.11 $$\begin{aligned} v = w_0 - \phi _n. \end{aligned}$$

Now we fix $n=5$, and study the Cauchy problem (2.8)–(2.10). For this we need a convenient functional setup.

## Functional Setup

This section is devoted to defining the function spaces in which we study the Cauchy evolution of (2.8). Furthermore, we gather the basic embedding properties that will be used later on.

### The Space $X^k$

Let $k \in {\mathbb {N}}$ and $f \in C^{\infty }_{c,\textrm{rad }}({\mathbb {R}}^5)$. As usual, we define the Sobolev norm $\Vert f \Vert _{\dot{H}^{k}({\mathbb {R}}^5)}:= \Vert |\cdot |^{k} {\mathcal {F}} f \Vert _{L^2({\mathbb {R}}^5)}$ via the Fourier transform. Since we are concerned with radial functions only, i.e., $f = {{\tilde{f}}}(|\cdot |)$, we straightforwardly get that

$$\begin{aligned} \Vert f \Vert _{\dot{H}^{k}({\mathbb {R}}^5)} = \Vert D^{k} f\Vert _{L^2({\mathbb {R}}^5)}, \end{aligned}$$

with

$$\begin{aligned} D^k f := {\left\{ \begin{array}{ll} \Delta ^{\frac{k}{2}}_{\textrm{rad}} {{\tilde{f}}}(|\cdot |), &{} \text {for k even,} \\ \big (\Delta ^{\frac{k-1}{2}}_{\textrm{rad}} {{\tilde{f}}} \big )'(|\cdot |), &{} \text {for k odd,} \end{array}\right. } \end{aligned}$$

where $\Delta _{\textrm{rad}}:= r^{-4} \partial _r ( r^{4} \partial _r)$ denotes the radial Laplace operator on ${\mathbb {R}}^5$. We also define an inner product on $C^{\infty }_{c,\textrm{rad }}({\mathbb {R}}^5)$ by

$$\begin{aligned} \langle f,g\rangle _{X^k}:= \langle D f, D g\rangle _{L^2({\mathbb {R}}^5)} + \langle D^k f, D^k g\rangle _{L^2({\mathbb {R}}^5)}, \end{aligned}$$

with corresponding norm $\Vert f \Vert _{X^k}:= \sqrt{\langle f,f\rangle _{X^k}}$. The central space of our analysis is the completion of $(C^{\infty }_{c,\textrm{rad }}({\mathbb {R}}^5), \Vert \cdot \Vert _{X^k})$, and we denote it by $X^{k}$. Throughout the paper we frequently use the equivalence

$$\begin{aligned} \Vert f \Vert ^2_{X^k} = \Vert f\Vert ^2_{\dot{H}^1({\mathbb {R}}^5)} + \Vert f\Vert ^2_{\dot{H}^k({\mathbb {R}}^5)} \simeq \sum _{|\alpha | = 1} \Vert \partial ^{\alpha } f \Vert ^2_{L^2({\mathbb {R}}^5)} + \sum _{|\alpha | = k} \Vert \partial ^{\alpha } f \Vert ^2_{L^2({\mathbb {R}}^5)}. \end{aligned}$$

Now, we list several properties of $X^k$ that will be used later on. First, a simple application of the Fourier transform yields the following interpolation inequality:

#### Lemma 3.1

Let $$k \in {\mathbb {N}}$$. Then

$$\begin{aligned} \Vert \partial ^\alpha f \Vert _{L^2({\mathbb {R}}^5)} \lesssim \Vert f \Vert _{X^k} \end{aligned}$$

for all $f \in C^{\infty }_{c,\textrm{rad }}({\mathbb {R}}^5)$, and all $\alpha \in {\mathbb {N}}_0^5$ with $1 \leqq |\alpha |\leqq k.$

Also, it is straightforward to see that the following result holds:

#### Lemma 3.2

Let $$k \in {\mathbb {N}}$$, $$k \geqq 3$$. Then

$$\begin{aligned} X^{k} \hookrightarrow W_{\textrm{rad}}^{{k-3},\infty }({\mathbb {R}}^5). \end{aligned}$$

In particular, elements of $X^k$ can be identified with functions in $C_\textrm{rad }^{k-3}({\mathbb {R}}^5)$. Furthermore, $X^{k}$ is a Banach algebra, i.e.,

$$\begin{aligned} \Vert f g\Vert _{X^k} \lesssim \Vert f \Vert _{X^k} \Vert g \Vert _{X^k} \end{aligned}$$

for all $f,g \in X^{k}$. In addition, $X^{k_2}$ embeds continuously into $X^{k_1}$ for $k_1 \leqq k_2$; shortly

3.1 $$\begin{aligned} X^{k_2}\hookrightarrow X^{k_1}. \end{aligned}$$

The next result follows from an elementary approximation argument.

#### Lemma 3.3

Let $$k \in {\mathbb {N}}$$, $$k \geqq 3$$. Define

$$\begin{aligned} {\mathcal {C}}:= \{ f \in C_{rad }^{\infty }({\mathbb {R}}^5): \text {for all } \kappa \in {\mathbb {N}}_0, \, |D^{\kappa } f(x)| \lesssim \langle x \rangle ^{-2-\kappa } \}. \end{aligned}$$

Then $${\mathcal {C}} \subset X^{k} $$.

Throughout the paper, we frequently use the commutator relation

3.2 $$\begin{aligned} \partial ^{\alpha } \Lambda = \Lambda \partial ^{\alpha } + k \partial ^{\alpha }, \end{aligned}$$

for $\alpha \in {\mathbb {N}}_0$, $|\alpha | = k$, as well as its radial analogue

3.3 $$\begin{aligned} D^k \Lambda = \Lambda D^k + k D^k. \end{aligned}$$

### Weighted $L^2-$Spaces

For $x \in {\mathbb {R}}^5$, we set

$$\begin{aligned} \sigma _0(x):= e^{-|x|^2/4} \quad \text {and} \quad \sigma (x):= \phi (x)^{-2} e^{-|x|^2/4} \end{aligned}$$

where

$$\begin{aligned}\phi (x):= \phi _5(x) = \frac{2}{2+|x|^2}.\end{aligned}$$

If for a non-negative measurable function $\omega$ on ${\mathbb {R}}^5$ we denote

$$\begin{aligned} L^2_{\omega }({\mathbb {R}}^5):=\{ f:{\mathbb {R}}^5 \rightarrow {\mathbb {C}}: f \text { is measurable and } \int _{{\mathbb {R}}^5}|f|^2w < \infty \}, \end{aligned}$$

then we define the following Hilbert spaces of radial functions

$$\begin{aligned} {\mathcal {H}}_0:= \{ f \in L^2_{\sigma _0}({\mathbb {R}}^5): f\text { is radial} \}, \quad {\mathcal {H}}:= \{ f \in L^2_{\sigma }({\mathbb {R}}^5): f\text { is radial} \}, \end{aligned}$$

with the corresponding inner products

$$\begin{aligned} \langle f,g \rangle _{{\mathcal {H}}_0} = \int _{{\mathbb {R}}^5} f(x) \overline{g(x)} \sigma _0(x) \textrm{d}x, \quad \langle f,g \rangle _{{\mathcal {H}}} = \int _{{\mathbb {R}}^5} f(x) \overline{g(x)} \sigma (x) \textrm{d}x. \end{aligned}$$

We note that both ${\mathcal {H}}_0$ and ${\mathcal {H}}$ have $C^{\infty }_{c,\textrm{rad}}({\mathbb {R}}^5)$ as a dense subset. An immediate consequence of the exponential decay of the weight functions is the following result:

#### Lemma 3.4

Let $$k \in {\mathbb {N}}, k \geqq 3$$. Then

$$\begin{aligned} \Vert f \Vert _{{\mathcal {H}}_0} \lesssim \Vert f \Vert _{{\mathcal {H}}} \lesssim \Vert f \Vert _{L^{\infty }({\mathbb {R}}^5)} \lesssim \Vert f \Vert _{X^k} \end{aligned}$$

for every $f \in C^{\infty }_{c,\textrm{rad}}({\mathbb {R}}^5)$. Consequently, we have the following continuous embeddings

$$\begin{aligned} X^k \hookrightarrow {\mathcal {H}} \hookrightarrow {\mathcal {H}}_0. \end{aligned}$$

### Operators

For convenience, we copy here the linear operators defined in (2.7) and (2.9)

3.4 $$\begin{aligned} L_0 f:= \Delta f - \frac{1}{2} \Lambda f - f, ~ L' f:= 2\Lambda (\phi f)+12\phi f, ~ N(f):= \Lambda f^2 + 6 f^2, \nonumber \\ \end{aligned}$$

and we formally let

$$\begin{aligned} L:=L_0+L'. \end{aligned}$$

Note that by Lemma 3.3, $\phi \in X^{k}$ for any $k \in {\mathbb {N}}, k \geqq 3$, and the same holds for $\Lambda \phi$.

## Linear Theory

In this section we concentrate on the linear version of (2.8), and show that it is well-posed in $X^k$ for $k \geqq 3$. To accomplish this, we use semigroup theory. Before we state the central result of the section, we make some technical preparations. First, we note that due to the underlying time-translation symmetry, the linear operator *L* has a formal unstable eigenvalue, $\lambda =1$, with an explicit eigenfunction

4.1 $$\begin{aligned} \nu (x):= \phi (x) + \frac{1}{2} \Lambda \phi (x) = \frac{4}{(2+|x|^2)^2}. \end{aligned}$$

According to Lemma 3.3, the function $\nu$ belongs to $X^k$, and this allows us to define a projection operator ${\mathcal {P}}: X^k \rightarrow X^k$ by

4.2 $$\begin{aligned} {\mathcal {P}} f:= \langle f, g \rangle _{{\mathcal {H}}} ~ g, \quad \text {where} \quad g = \frac{\nu }{\Vert \nu \Vert _{{\mathcal {H}}}}. \end{aligned}$$

This whole section is devoted to proving the next theorem, which, in short, states that the linear flow of (2.8) decays exponentially in time on the kernel of ${\mathcal {P}}$.

### Theorem 4.1

Let $k \in {\mathbb {N}}$, $k \geqq 3$. Then the operator $L: C^{\infty }_{c,\textrm{rad }}({\mathbb {R}}^5) \subset X^k \rightarrow X^k$ is closable, and its closure generates a strongly continuous semigroup $(S(\tau ))_{\tau \geqq 0}$ of bounded operators on $X^k$. Furthermore, there exists $\omega _k \in (0,\frac{1}{4})$ such that

4.3 $$\begin{aligned} \Vert S(\tau ) (1- {\mathcal {P}}) f \Vert _{X^k} \lesssim e^{-\omega _{k} \tau } \Vert (1- {\mathcal {P}}) f \Vert _{X^k} \quad \text {and} \quad S(\tau ){\mathcal {P}} f = e^{\tau } {\mathcal {P}} f, \end{aligned}$$

for all $f \in X^k$ and all $\tau \geqq 0$.

The mere fact that the closure of *L* generates a semigroup on $X^k$ can be proved by an application of the Lumer–Phillips Theorem, see Section  4.2. However, determining the precise growth of the semigroup is highly non-trivial, in view of the non-self-adjoint nature of the problem and the lack of an abstract spectral mapping theorem that would apply to this situation. To get around this issue, we combine the analysis in $X^{k}$ with the self-adjoint theory for *L* in ${\mathcal {H}}$.

### The Linearized Evolution on ${\mathcal {H}}$

We equip $L_0$ and *L* with domains

4.4 $$\begin{aligned} {\mathcal {D}}(L_0)={\mathcal {D}}(L):=C^{\infty }_{c,\textrm{rad }}({\mathbb {R}}^5). \end{aligned}$$

The following result for the operator $L_0$ is well-known, and we refer the reader to [28], Lemma 3.1, for a detailed proof:

#### Lemma 4.2

The operator $L_0: {\mathcal {D}}(L_0) \subset {\mathcal {H}}_0 \rightarrow {\mathcal {H}}_0$ is closable, and its closure $({\mathcal {L}}_0,{\mathcal {D}}({\mathcal {L}}_0))$ generates a strongly continuous semigroup $(S_0(\tau ))_{\tau \geqq 0}$ of bounded operators on ${\mathcal {H}}_0$. Explicitly,

$$\begin{aligned}{}[S_0(\tau )f](x)=e^{-\tau }(G_\tau *f)(e^{-\tau /2}x), \end{aligned}$$

where $$G_\tau (x)=[4\pi \alpha (\tau )]^{-\frac{5}{2}}e^{-|x|^2/4\alpha (\tau )}$$ and $$\alpha (\tau )=1-e^{-\tau }$$.

The operator *L*, on the other hand, has a self-adjoint realization in the space ${\mathcal {H}}$; we have the following fundamental result.

#### Proposition 4.3

The operator $L: {\mathcal {D}}(L) \subset {\mathcal {H}} \rightarrow {\mathcal {H}}$ is closable, and its closure $({\mathcal {L}},{\mathcal {D}}({\mathcal {L}}))$ generates a strongly continuous semigroup $(S(\tau ))_{\tau \geqq 0}$ of bounded operators on ${\mathcal {H}}$. The spectrum of ${\mathcal {L}}$ consists of a discrete set of eigenvalues, and moreover,

4.5 $$\begin{aligned} \sigma ({\mathcal {L}}) \subset (-\infty ,0) \cup \{1\}, \end{aligned}$$

where $\lambda =1$ is a simple eigenvalue with the normalized eigenfunction *g* from (4.2). Furthermore, for the orthogonal projection ${\mathcal {P}}: {\mathcal {H}} \rightarrow {\mathcal {H}}$ defined in (4.2) there exists $\omega _0 >0$ such that

4.6 $$\begin{aligned} \Vert S(\tau ) (1- {\mathcal {P}}) f \Vert _{{\mathcal {H}} } \leqq e^{-\omega _0 \tau } \Vert (1- {\mathcal {P}}) f \Vert _{{\mathcal {H}}} \quad \text {and} \quad S(\tau ) {\mathcal {P}} f = e^{\tau } {\mathcal {P}}f, \end{aligned}$$

for all $f \in {\mathcal {H}}$ and all $\tau \geqq 0$.

#### Proof

We define the unitary map

$$\begin{aligned} U: L^2({\mathbb {R}}^+) \rightarrow {\mathcal {H}}, \quad u \mapsto Uu = |{\mathbb {S}}^{4}|^{-\frac{1}{2}} |\cdot |^{-2} e^{\frac{|\cdot |^2}{8}} \phi (|\cdot |) u(|\cdot |) \end{aligned}$$

and note that $-L = U A U^{-1}$ with

$$\begin{aligned} A u(r) = - u''(r) + q(r) u(r) \end{aligned}$$

where

$$\begin{aligned} q(r):= \frac{2}{r^2} + \frac{r^2}{16} - \frac{5}{4} -\frac{16}{(2+r^2)^2}-\frac{4}{2+r^2}, \end{aligned}$$

and ${\mathcal {D}}(A) = U^{-1} {\mathcal {D}}(L)$. Let us denote by $A_c$ the restriction of *A* to $C^{\infty }_c({\mathbb {R}}^+)$. Using standard results, we infer that $A_c$ is limit-point at both endpoints of the interval $(0,\infty )$ (see, e.g., [54], Theorem 6.6, p. 96, and Theorem 6.4, p. 91). Hence, the unique self-adjoint extension of $A_c$ is given by its closure, which is the maximal operator ${\mathcal {A}}: {\mathcal {D}}({\mathcal {A}}) \subset L^2({\mathbb {R}}^+) \rightarrow L^2({\mathbb {R}}^+)$, where

$$\begin{aligned} {\mathcal {D}}({\mathcal {A}}):= \{ u \in L^2({\mathbb {R}}^+): u,u' \in AC_{\textrm{loc}}({\mathbb {R}}^+), A u \in L^2({\mathbb {R}}^+) \}, \end{aligned}$$

and ${\mathcal {A}} u = A u$ for $u \in {\mathcal {D}}({\mathcal {A}})$. The inclusion $A_c \subset A \subset {\mathcal {A}} = \overline{A_c}$ implies ${\overline{A}} ={\mathcal {A}}$. Now, since *q* is bounded from below, the same is true for the operator ${\mathcal {A}}$, i.e, there is a constant $\mu > 0$ such that $\textrm{Re}\langle {\mathcal {A}} u, u \rangle _{L^2({\mathbb {R}}^+)} \geqq -\mu \Vert u \Vert _{L^2({\mathbb {R}}^+)}$ for all $u \in {\mathcal {D}}({\mathcal {A}})$. Since $q(r) \rightarrow \infty$ when $r \rightarrow \infty$, ${\mathcal {A}}$ has compact resolvent, and its spectrum therefore consists of a discrete set of eigenvalues. The analogous properties of *L* follow by the unitary equivalence. In particular, $L: {\mathcal {D}}(L) \subset {\mathcal {H}} \rightarrow {\mathcal {H}}$ is essentially self-adjoint and its closure is given by ${\mathcal {L}} = - U {\mathcal {A}} U^{-1}$ with ${\mathcal {D}}({\mathcal {L}}) = U {\mathcal {D}}({\mathcal {A}})$. Moreover, we have that $\textrm{Re}\langle {\mathcal {L}} f, f \rangle _{{\mathcal {H}}} \leqq \mu \Vert f \Vert _{{\mathcal {H}}}$ for all $f \in {\mathcal {D}}({\mathcal {L}})$. This implies that ${\mathcal {L}}$, being self-adjoint, generates a strongly continuous semigroup on ${\mathcal {H}}$.

Next, we describe the spectral properties of $${\mathcal {L}}$$. Obviously, $$g \in {\mathcal {D}}({\mathcal {L}})$$ and $$ L g = g$$ by explicit calculation. Hence, $${\tilde{g}}:= U^{-1} g $$ satisfies $$(1 + A) {\tilde{g}} = 0$$. Moreover, $${\tilde{g}}$$ is strictly positive on $$(0, \infty )$$. Hence, we have the factorization $$A = A^{-} A^{+}-1$$, where

$$\begin{aligned} A^{\pm } = \pm \partial _r - \frac{ {\tilde{g}}'(r)}{{\tilde{g}}(r)}. \end{aligned}$$

We define $A_{{\mathcal {S}}}:= A^{+} A^{-} - 1$. Explicitly, we have

$$\begin{aligned} A_{{\mathcal {S}}} u(r) = - u''(r) + \frac{6}{r^2} u(r) + Q_S(r) u(r), \end{aligned}$$

with

$$\begin{aligned} Q_S(r):= \frac{r^2}{16}-\frac{3}{4} - \frac{8}{2+r^2}. \end{aligned}$$

This gives rise to the maximally defined self-adjoint operator ${\mathcal {A}}_{{\mathcal {S}}}: {\mathcal {D}}({\mathcal {A}}_{{\mathcal {S}}}) \subset L^2({\mathbb {R}}^+) \rightarrow L^2({\mathbb {R}}^+)$ which is, by construction, isospectral to ${\mathcal {A}}$ except for $\lambda = -1$. For a detailed discussion on the above process of “removing” an eigenvalue, see [29], Section B.1. Now, to prove (4.5), it is enough to show that ${\mathcal {A}}_S$ does not have any eigenvalues in $(-\infty ,0]$. For this, we use a so-called GGMT criterion, see e.g. Appendix A in [16], that we adapted to our problem in [28], Theorem A.1. In particular, we show that for $p=2$,

4.7 $$\begin{aligned} \int _0^{\infty } r^{2p-1} |Q_S^-(r)|^p \textrm{d}r <\frac{(4\alpha +1)^{\frac{2p-1}{2}}p^p \Gamma (p)^2}{(p-1)^{p-1}\Gamma (2p)} \end{aligned}$$

where $\alpha = 6$ and $Q_S^-(r) = \min \{ Q_S(r), 0 \}$. By calculating explicitly the root of $Q_S$ one can easily check that $Q_S(r) > 0$ for $r \geqq 5$, hence

$$\begin{aligned} \int _0^{\infty } r^{3} Q_S^-(r)^2 \textrm{d}r < \int _0^{5} r^{3} Q_S(r)^2 \textrm{d}r. \end{aligned}$$

By noting that

$$\begin{aligned} r^3 Q_S(r)^2 = \frac{r^7}{256}-\frac{r^5}{r^2+2}-\frac{3 r^5}{32} +\frac{9 r^3}{16} +\frac{12 r^3}{r^2+2}+\frac{64 r^3}{\left( r^2+2\right) ^2}, \end{aligned}$$

we can calculate the integral explicitly and find that

$$\begin{aligned} \int _0^{5} r^{3} Q_S(r)^2 \textrm{d}r = \frac{1305275}{55296}-18 \ln (2)+18 \ln (27) < \frac{250}{3} \end{aligned}$$

where the right-hand side of this inequality is the value of the right-hand side of Equation (4.7) for $p=2$ and $\alpha =6$. Theorem A.1 in [28] now implies that $\sigma ({\mathcal {A}}_S) \subset (0,+\infty )$, and (4.5) follows. Furthermore, a simple ODE analysis yields that the geometric eigenspace of $\lambda =1$ is equal to $\langle g \rangle$. Consequently, ${\mathcal {P}}$ is the orthogonal projection onto ${\text {rg}}{\mathcal {P}}$ in ${\mathcal {H}}$, and we readily get (4.6). $\square$

The next lemma shows that the growth bounds are preserved when the linear evolution is measured in graph norms associated to fractional powers of the operator $1- {\mathcal {L}}$. This result will be crucial in Sect. 4.2, in particular, in conjunction with Lemma 4.6 below.

#### Lemma 4.4

There is a unique self-adjoint, positive operator $(1 - {\mathcal {L}})^{\frac{1}{2}}$ with $C^\infty _{c,\textrm{rad}}({\mathbb {R}}^5)$ as a core, such that $\big ((1 - {\mathcal {L}})^{\frac{1}{2}}\big )^2 = 1 - {\mathcal {L}}$. For $k \in {\mathbb {N}}_0$ and $f \in {\mathcal {D}}((1- {\mathcal {L}})^{k/2})$ we define the graph norm

$$\begin{aligned} \Vert f\Vert _{{\mathcal {G}}((1- {\mathcal {L}})^{k/2})} := \Vert f \Vert _{{\mathcal {H}}} + \Vert (1- {\mathcal {L}})^{k/2} f \Vert _{{\mathcal {H}}}, \end{aligned}$$

and infer that

$$\begin{aligned} \Vert S(\tau ) (1 -{\mathcal {P}}) f \Vert _{{\mathcal {G}}((1- {\mathcal {L}})^{k/2})} \leqq e^{-\omega _0 \tau } \Vert (1 - {\mathcal {P}}) f \Vert _{{\mathcal {G}}((1- {\mathcal {L}})^{k/2})} \end{aligned}$$

for every $k \in {\mathbb {N}}_0$, $f \in {\mathcal {D}}((1- {\mathcal {L}})^{k/2})$ and all $\tau \geqq 0$.

#### Proof

By standard results (see, e.g., [38], p. 281, Theorem 3.35), the square root $(1 - {\mathcal {L}})^{\frac{1}{2}}$ exists and commutes with any bounded operator that commutes with ${\mathcal {L}}$. We show that $C^\infty _{c,\textrm{rad}}({\mathbb {R}}^5)$ is a core of $(1-{\mathcal {L}})^\frac{1}{2}$. Let $\varepsilon > 0$. Since ${\mathcal {D}}({\mathcal {L}})$ is core for $(1 - {\mathcal {L}})^{\frac{1}{2}}$ and $C^\infty _{c,\textrm{rad}}({\mathbb {R}}^5)$ is a core for $1 - {\mathcal {L}}$ there is ${{\tilde{f}}} \in C^\infty _{c,\textrm{rad}}({\mathbb {R}}^5)$ such that $\Vert f - {{\tilde{f}}} \Vert _{{\mathcal {H}}} + \Vert (1- {\mathcal {L}})^{\frac{1}{2}} (f - {{\tilde{f}}}) \Vert _{{\mathcal {H}}} < \varepsilon$, by using that $\Vert (1- {\mathcal {L}})^{\frac{1}{2}} f\Vert _{{\mathcal {H}}} \lesssim \Vert (1- {\mathcal {L}})f\Vert _{{\mathcal {H}}} + \Vert f \Vert _{{\mathcal {H}}}$. The growth bounds for the semigroup can be proved by induction using the fact that $(1-{\mathcal {L}})^{\frac{1}{2}}$ commutes with the projection and the semigroup. $\square$

We conclude this section by proving two technical results that will be crucial in the sequel.

#### Lemma 4.5

Let $k \in {\mathbb {N}}_0$ and $R >0$. Then

$$\begin{aligned} \Vert \partial ^{\alpha } f \Vert _{L^2({\mathbb {B}}^5_R)} \lesssim \sum _{j=0}^{k} \Vert f \Vert _{{\mathcal {G}}((1-{\mathcal {L}})^{j/2})}, \end{aligned}$$

for all $f \in C^{\infty }_{c,\textrm{rad}}({\mathbb {R}}^5)$ and all $\alpha \in {\mathbb {N}}_0^5$ with $|\alpha | = k$.

#### Proof

First, for $f \in C^{\infty }_{c,\textrm{rad}}({\mathbb {R}}^5)$ and $\mu (x):= |x|^4 e^{-|x|^2/4 }$ we define

$$\begin{aligned} Bf(x) := \phi (x)^{2} \frac{\textrm{d}}{\textrm{d}|x|} \left( \phi (x)^{-2} f(x) \right) , \quad B^*f(x) := - \mu (x)^{-1} \frac{\textrm{d}}{\textrm{d}|x|} \left( \mu (x) f(x) \right) . \end{aligned}$$

By inspection, it follows that $1 - L = B^* B$ and thus,

$$\begin{aligned} \Vert (1-{\mathcal {L}})^{\frac{1}{2}} f \Vert ^2_{{\mathcal {H}}} = \langle (1-{\mathcal {L}}) f,f \rangle _{{\mathcal {H}}} = \langle B^*B f, f \rangle _{{\mathcal {H}}} = \langle B f, B f \rangle _{{\mathcal {H}}} = \Vert B f\Vert ^2_{{\mathcal {H}}}, \end{aligned}$$

where the fact that *B* and $B^*$ are formally adjoint follows from a straightforward calculation. Hence,

$$\begin{aligned} \Vert (1-{\mathcal {L}})^{\frac{1}{2}} f \Vert _{{\mathcal {H}}} = \Vert B f\Vert _{{\mathcal {H}}} \end{aligned}$$

for $f \in C^{\infty }_{c,\textrm{rad}}({\mathbb {R}}^5)$. With this at hand, we prove the lemma by induction. For $k =0$ the inequality is immediate, and for $k=1$ we have that

$$\begin{aligned} \Vert \partial _i f \Vert _{L^2({\mathbb {B}}^5_R)} \lesssim \Vert {{\tilde{f}}}'(|\cdot |) \Vert _{L^2(0,R)} \lesssim \Vert Bf\Vert _{{\mathcal {H}}} + \Vert f \Vert _{{\mathcal {H}}} = \Vert (1-{\mathcal {L}})^{\frac{1}{2}} f \Vert _{{\mathcal {H}}} + \Vert f \Vert _{{\mathcal {H}}}. \end{aligned}$$

Assume that the claim holds up to some $k \geqq 1$. Then we have that for all $1 \leqq j \leqq k$

$$\begin{aligned} \Vert D^{j+1} f\Vert _{L^2({\mathbb {B}}^5_R)}&= \Vert D^{j-1}D^2f \Vert _{L^2({\mathbb {B}}^5_R)} \lesssim \Vert D^{j-1}(1-{\mathcal {L}})f \Vert _{L^2({\mathbb {B}}^5_R)}\\&\quad + \Vert D^{j-1}f \Vert _{L^2({\mathbb {B}}^5_R)} + \Vert D^{j-1}\Lambda f \Vert _{L^2({\mathbb {B}}^5_R)}\\&\quad + \Vert D^{j-1}\Lambda (\phi f) \Vert _{L^2({\mathbb {B}}^5_R)} + \Vert D^{j-1}(\phi f) \Vert _{L^2({\mathbb {B}}^5_R)} \\&\lesssim \sum _{|\beta |\leqq j-1}\Vert \partial ^{\beta } (1- {\mathcal {L}})f \Vert _{L^2({\mathbb {B}}^5_R)} + \sum _{|\beta |\leqq j} \Vert \partial ^\beta f \Vert _{L^2({\mathbb {B}}^5_R)} \\&\lesssim \sum _{i=0}^{j-1}\Vert (1- {\mathcal {L}})f \Vert _{{\mathcal {G}}((1-{\mathcal {L}})^{i/2})} + \sum _{i=0}^{j}\Vert f \Vert _{{\mathcal {G}}((1-{\mathcal {L}})^{i/2})} \\&\lesssim \sum _{i=0}^{j+1}\Vert f \Vert _{{\mathcal {G}}((1-{\mathcal {L}})^{i/2})}. \end{aligned}$$

Lemma A.1 then implies the claim for $|\alpha |=k+1$. $\square$

Finally, we show that graph norms can be controlled by $X^k$-norms.

#### Lemma 4.6

Let $$k \in {\mathbb {N}}$$, $$k \geqq 3$$. Then

$$\begin{aligned} \Vert (1-{\mathcal {L}})^{\kappa /2} f \Vert _{{\mathcal {H}}} \lesssim \Vert f \Vert _{X^k} \end{aligned}$$

for all $f \in X^k$ and all $\kappa \in \{0, \dots , k \}$.

#### Proof

We prove the statement for $f \in C^{\infty }_{c,\textrm{rad}}({\mathbb {R}}^5)$. The claim then follows by density and the closedness of $(1-{\mathcal {L}})^{\kappa /2}$. First, it is easy to see that

$$\begin{aligned} \Vert (1 -{\mathcal {L}})^{\kappa /2} f \Vert _{{\mathcal {H}}} \lesssim \sum _{|\alpha |\leqq \kappa } \Vert p_\alpha \partial ^\alpha f \Vert _{{\mathcal {H}}} \end{aligned}$$

for smooth, radial and polynomially bounded functions $p_\alpha$. Using the exponential decay of the weight function $\sigma$ along with interpolation, see Lemma 3.1, and Hardy’s inequality, we get

$$\begin{aligned} \Vert (1 -{\mathcal {L}})^{\kappa /2} f \Vert _{{\mathcal {H}}}&\lesssim \sum _{1\leqq |\alpha | \leqq \kappa } \Vert \partial ^\alpha f \Vert _{L^2({\mathbb {R}}^5)} + \Vert |\cdot |^{-1} f \Vert _{L^2({\mathbb {R}}^5)} \lesssim \Vert f \Vert _{X^k}. \end{aligned}$$

$$\square $$

### The Linearized Evolution on $X^k$

With the technical results from above, we now show that the linear evolution of (2.8) is well-posed in $X^k$. Since $C^{\infty }_{c,\textrm{rad}}({\mathbb {R}}^5)$ is dense in $X^{k}$, we consider *L* as defined in (4.4).

#### Proposition 4.7

Let $k \in {\mathbb {N}}$, $k \geqq 3$. The operator $L: {\mathcal {D}}(L) \subset X^k \rightarrow X^k$ is closable and with $({\mathcal {L}}_k, {\mathcal {D}}({\mathcal {L}}_k))$ denoting the closure we have that ${\mathcal {C}} \subset {\mathcal {D}}({\mathcal {L}}_k)$. Furthermore, the operator ${\mathcal {L}}_k$ generates a strongly continuous semigroup $(S_{k}(\tau ))_{\tau \geqq 0}$ of bounded operators on $X^k$, which coincides with the restriction of $S(\tau )$ to $X^k$, i.e,

$$\begin{aligned} S_{k}(\tau )= S(\tau )|_{X^{k}} \end{aligned}$$

for all $$\tau \geqq 0$$.

#### Proof

We prove the first part of the statement by an application of the Lumer-Phillips theorem. For this, we show that

4.8 $$\begin{aligned} {\text {Re}}\langle L f,f\rangle _{X^k} \leqq {\bar{\omega }}_k \Vert f \Vert ^2_{X^k} \end{aligned}$$

for some ${\bar{\omega }}_k >0$ and all $f \in {\mathcal {D}}(L)$. In fact, we prove a more general estimate, which will be instrumental in proving Theorem 4.1 later on. More precisely, we show that for $R \geqq 1$, there are constants $C_k, C_{R,k} > 0$ such that for all $f \in C^{\infty }_{c,\textrm{rad}}({\mathbb {R}}^5)$,

4.9 $$\begin{aligned} {\text {Re}}\langle L f, f \rangle _{X^k} \leqq (- \tfrac{1}{4} + \tfrac{C_k}{R^2} )\Vert f \Vert ^2_{X^k} + C_{R,k} \sum _{j=0}^{k} \Vert f \Vert ^2_{{\mathcal {G}}((1- {\mathcal {L}})^{j/2})}. \end{aligned}$$

An application of Lemma 4.6 to Equation (4.9) immediately implies Equation (4.8). Equation (4.9) will be proved in several steps. First, recall that $L = L_0 + L'$. By partial integration,

4.10 $$\begin{aligned} \begin{aligned} \langle D^k \Delta f, D^k f \rangle _{L^2({\mathbb {R}}^5)}&=\langle D^{k+2} f, D^k f\rangle _{L^2({\mathbb {R}}^5)} = -\Vert D^{k+1} f\Vert ^2_{L^2({\mathbb {R}}^5)}. \end{aligned} \end{aligned}$$

Based on the identity

$$\begin{aligned} {\text {Re}}\langle \Lambda f,f \rangle _{L^2({\mathbb {R}}^5)} = -\tfrac{5}{2} \Vert f\Vert ^2_{L^2({\mathbb {R}}^5)}, \end{aligned}$$

which easily follows from integration by parts and the commutator relation Equation (3.3), we get

4.11 $$\begin{aligned} {\text {Re}}\langle D^{k} \Lambda f,D^{k} f \rangle _{L^2({\mathbb {R}}^5)} = (k -\tfrac{5}{2}) \Vert D^{k} f \Vert ^2_{L^2({\mathbb {R}}^5)}. \end{aligned}$$

Consequently,

4.12 $$\begin{aligned} {\text {Re}}\langle L_0 f, f \rangle _{X^k} \leqq - \tfrac{1}{4} \Vert f \Vert ^2_{X^k} \end{aligned}$$

and thus

$$\begin{aligned} {\text {Re}}\langle L f, f \rangle _{X^k} = {\text {Re}}\langle (L_0 + L') f, f \rangle _{X^k} \leqq - \tfrac{1}{4} \Vert f \Vert ^2_{X^k} + {\text {Re}}\langle L' f, f \rangle _{X^k}. \end{aligned}$$

To obtain Equation (4.8), one can estimate the part containing $L'$ in $X^k$ in a straightforward manner. However, for the refined bound (4.9), the argument is more involved. We write

4.13 $$\begin{aligned} {\text {Re}}\langle L' f, f \rangle _{X^k} = {\text {Re}}\langle V f, f \rangle _{X^k} + 2 {\text {Re}}\langle \phi \Lambda f, f \rangle _{X^k} \end{aligned}$$

with

$$\begin{aligned} V(x):= 2 x \cdot \nabla \phi (x) + 12 \phi (x). \end{aligned}$$

Note that $V \in C^{\infty }_{\textrm{rad}}({\mathbb {R}}^5)$ and the properties of $\phi$ imply that

4.14 $$\begin{aligned} |\partial ^{\alpha } V(x)| \lesssim \langle x \rangle ^{-2-|\alpha |}, \end{aligned}$$

for $\alpha \in {\mathbb {N}}_0^5$. First, we prove that

4.15 $$\begin{aligned} {\text {Re}}\langle D^k (V f), D^k f \rangle _{L^2({\mathbb {R}}^5)} \leqq C_{R} \sum _{j=0}^{k} \Vert f \Vert ^2_{{\mathcal {G}}((1-{\mathcal {L}})^{j/2})} + \tfrac{C}{R^2} \Vert f \Vert ^2_{X^k} \end{aligned}$$

for suitable constants $C_{R}, C >0$. For this, we use the fact that

$$\begin{aligned} {\text {Re}}\langle D^k (V f), D^k f \rangle _{L^2({\mathbb {R}}^5)} \lesssim \sum _{|{\tilde{\alpha }}|=|\alpha | = k } | {\text {Re}}\langle \partial ^{{\tilde{\alpha }}} (V f), \partial ^{\alpha } f \rangle _{L^2({\mathbb {R}}^5)}|, \end{aligned}$$

for all $f \in C^{\infty }_{c,\textrm{rad}}({\mathbb {R}}^5)$. Hence, we can apply the standard Leibniz rule to estimate products. More precisely, for $\alpha ,\beta ,\gamma \in {\mathbb {N}}_0^5$ for which $|\alpha |=|\beta +\gamma | = k$, we estimate

$$\begin{aligned} |\langle \partial ^{\beta } V \partial ^{\gamma } f, \partial ^{\alpha } f \rangle _{L^2({\mathbb {R}}^5)}| \leqq \Vert |\partial ^{\beta } V|^{\frac{|\beta |+1}{|\beta |+2}} \partial ^{\gamma } f\Vert ^2_{L^2({\mathbb {R}}^5)} + \Vert |\partial ^{\beta } V|^{\frac{1}{|\beta |+2}} \partial ^{\alpha } f \Vert ^2_{L^2({\mathbb {R}}^5)}. \end{aligned}$$

By Equation (4.14) and Lemmas 4.5 and 3.1 the last term can be bounded by

$$\begin{aligned} \Vert |\partial ^{\beta } V|^{\frac{1}{|\beta |+2}} \partial ^{\alpha } f \Vert ^2_{L^2({\mathbb {R}}^5)}&\leqq C \Vert \partial ^{\alpha } f \Vert ^2_{L^2({\mathbb {B}}^5_R)} + \tfrac{C}{R^2} \Vert \partial ^{\alpha } f \Vert ^2_{L^2({\mathbb {R}}^5 \setminus {\mathbb {B}}^5_R)} \\&\leqq C_{R} \sum _{j=0}^{k} \Vert f \Vert ^2_{{\mathcal {G}}((1-{\mathcal {L}})^{j/2})} + \tfrac{C}{R^2} \Vert f \Vert ^2_{X^k}. \end{aligned}$$

Similarly, we have that

$$\begin{aligned} \Vert |\partial ^{\beta } V|^{\frac{|\beta |+1}{|\beta |+2}} \partial ^{\gamma } f\Vert ^2_{L^2({\mathbb {R}}^5)} \leqq C_{R} \sum _{j=0}^{|\gamma |} \Vert f \Vert _{{\mathcal {G}}((1-{\mathcal {L}})^{j/2})} + \tfrac{C}{R^2} \Vert |\cdot |^{-|\beta |} \partial ^{\gamma } f \Vert ^2_{L^2({\mathbb {R}}^5 \setminus {\mathbb {B}}^5_R)}. \end{aligned}$$

For $\gamma = 0$, we estimate the last term by Hardy’s inequality,

$$\begin{aligned} \Vert |\cdot |^{-k} f \Vert _{L^2({\mathbb {R}}^5 \setminus {\mathbb {B}}^5_R)}&\leqq \Vert |\cdot |^{-1} f \Vert _{L^2({\mathbb {R}}^5 \setminus {\mathbb {B}}^5_R) } \leqq \Vert |\cdot |^{-1} f \Vert _{L^2({\mathbb {R}}^5) }\\&\lesssim \Vert D f\Vert _{L^2({\mathbb {R}}^5)} \lesssim \Vert f \Vert _{X^k}. \end{aligned}$$

For $\gamma \ne 0$, by interpolation and equivalence of norms, see Lemma  (3.1), we obtain

$$\begin{aligned} \Vert |\cdot |^{-|\beta |} \partial ^{\gamma } f \Vert _{L^2({\mathbb {R}}^5 \setminus {\mathbb {B}}^5_R)} \leqq \Vert \partial ^{\gamma } f \Vert _{L^2({\mathbb {R}}^5 \setminus {\mathbb {B}}^5_R)} \lesssim \Vert f \Vert _{X^k}, \end{aligned}$$

which implies Equation (4.15). To estimate the second term in Equation (4.13), we use the relation

4.16 $$\begin{aligned} {\text {Re}}\langle D^k (\phi \Lambda f), D^k f \rangle _{L^2({\mathbb {R}}^5)}\lesssim & {} | {\text {Re}}\langle \phi \Lambda D^kf, D^k f \rangle _{L^2({\mathbb {R}}^5)}| \nonumber \\{} & {} + \sum _{\begin{array}{c} 1 \leqq |\beta |\leqq k \\ |\alpha | = k, \end{array}} | \langle \varphi _\beta \partial ^\beta f, \partial ^{\alpha } f \rangle _{L^2({\mathbb {R}}^5)}| \end{aligned}$$

with certain smooth functions $|\varphi _{\beta }(x)| \lesssim \langle x \rangle ^{-2}$. Note that, by partial integration, we have the identity

$$\begin{aligned} {\text {Re}}\, \langle \phi \Lambda D^kf,D^kf \rangle _{L^2({\mathbb {R}}^5)} = -\tfrac{5}{2}\langle \phi D^kf,D^kf\rangle _{L^2({\mathbb {R}}^5)} - \tfrac{1}{2}\langle (\Lambda \phi ) D^kf,D^kf \rangle _{L^2({\mathbb {R}}^5)}. \end{aligned}$$

Therefore, the first term in (4.16) can be estimated

$$\begin{aligned} | {\text {Re}}\langle \phi \Lambda D^k f, D^k f \rangle _{L^2({\mathbb {R}}^5)}| \lesssim \Vert \langle \cdot \rangle ^{-1} D^k f \Vert ^2_{L^2({\mathbb {R}}^5)}. \end{aligned}$$

For the second term we have

$$\begin{aligned} | \langle \varphi _{\beta } \partial ^{\beta } f, \partial ^{\alpha } f \rangle _{L^2({\mathbb {R}}^5)} | \lesssim \Vert \langle \cdot \rangle ^{-1} \partial ^{\beta } f \Vert ^2_{L^2({\mathbb {R}}^5)} + \Vert \langle \cdot \rangle ^{-1} \partial ^{\alpha } f \Vert ^2_{L^2({\mathbb {R}}^5)}. \end{aligned}$$

Based on this, similarly to above we infer that

4.17 $$\begin{aligned} {\text {Re}}\langle D^k (\phi \Lambda f), D^k f \rangle _{L^2({\mathbb {R}}^5)} \leqq C_R \sum _{j=0}^{k} \Vert f \Vert _{{\mathcal {G}}((1-{\mathcal {L}})^{j/2})} + \tfrac{C}{R^2} \Vert f \Vert ^2_{X^k}, \end{aligned}$$

for suitably chosen $C_R, C >0$. Using the same arguments, we arrive at (4.15) and (4.17) for *D* instead of $D^k$, and we hence get (4.9), and thereby (4.8) as well. From this, we infer that the operator *L* is closable (see, e.g., [20], p. 82, Proposition 3.14-(iv)), and that the closure ${\mathcal {L}}_k: {\mathcal {D}}({\mathcal {L}}_k) \subset X^k \rightarrow X^k$, satisfies

4.18 $$\begin{aligned} {\text {Re}}\langle {\mathcal {L}}_k f,f\rangle _{X^k} \leqq {\bar{\omega }}_k \Vert f \Vert ^2_{X^k} \end{aligned}$$

for all $f \in {\mathcal {D}}({\mathcal {L}}_k)$ and some suitable ${\bar{\omega }}_k > 0$.

Next, we prove that ${\mathcal {C}} \subset {\mathcal {D}}({\mathcal {L}}_k)$ by showing that for $f \in {\mathcal {C}}$ there is a sequence $(f_n) \subset C^{\infty }_{c,\textrm{rad}}({\mathbb {R}}^5)$, such that $f_n \rightarrow f$ and $L f_n \rightarrow v$ in $X^k$. Then, by definition of the closure, $f \in {\mathcal {D}}({\mathcal {L}}_k)$ and ${\mathcal {L}}_k f = v$. We set $f_n = \chi (\cdot /n) f$, where $\chi$ a smooth, radial cut-off function equal to one for $|x| \leqq 1$ and zero for $|x| \geqq 2$. It is easy to see that $(f_n) \subset C^{\infty }_{c,\textrm{rad}}({\mathbb {R}}^5)$ converges to *f* in $X^k$. More precisely, by exploiting the decay of *f*,

$$\begin{aligned} \Vert f_n - f \Vert _{L^{\infty }({\mathbb {R}}^5)} = \Vert f_n - f \Vert _{L^{\infty }({\mathbb {R}}^5\setminus {\mathbb {B}}^5_n)} \lesssim \Vert f \Vert _{L^{\infty }({\mathbb {R}}^5\setminus {\mathbb {B}}^5_n)} \lesssim n^{-2}, \end{aligned}$$

we have $f_n \rightarrow f$ in $L^{\infty }({\mathbb {R}}^5)$. Furthermore, for $m \leqq n$, $f_n - f_m = 0$ on ${\mathbb {B}}^5_m$ and thus

$$\begin{aligned} \Vert D(\chi _n f) - D(\chi _m f)\Vert _{L^2({\mathbb {R}}^5)} = \Vert D(\chi _n f) - D(\chi _m f)\Vert _{L^2({\mathbb {R}}^5 \setminus {\mathbb {B}}^5_m)}. \end{aligned}$$

We have

$$\begin{aligned} \Vert D(\chi _n f) \Vert _{L^2({\mathbb {R}}^5\setminus {\mathbb {B}}^5_m)} \lesssim \Vert |\cdot |^{-2} D\chi _n \Vert _{L^2({\mathbb {R}}^5\setminus {\mathbb {B}}^5_m)} + \Vert |\cdot |^{-3} \chi _n \Vert _{L^2({\mathbb {R}}^5\setminus {\mathbb {B}}^5_m)} \lesssim n^{-\frac{1}{2}}, \end{aligned}$$

and similarly $\Vert D^{k}(\chi _n f) \Vert _{L^2({\mathbb {R}}^5{\setminus } {\mathbb {B}}^5_m)} \lesssim n^{-\frac{1}{2}}$. Thus, $\Vert f_n - f_m \Vert _{X^k} \lesssim n^{-\frac{1}{2}} + m^{-\frac{1}{2}}$ and $(f_n)$ converges in $X^k$ to some limiting function which must be equal to *f* by the $L^{\infty }-$embedding and the uniqueness of limits.

Now, $\Lambda f \in {\mathcal {C}}$ for $f \in {\mathcal {C}}$. Since $\Lambda f_n = f \Lambda \chi _n + \chi _n \Lambda f$ and

$$\begin{aligned} \Vert \Lambda f_n - \Lambda f \Vert _{L^{\infty }({\mathbb {R}}^5)} \lesssim \Vert \chi _n \Lambda f - \Lambda f \Vert _{L^{\infty }({\mathbb {R}}^5\setminus {\mathbb {B}}_n^5)} + \Vert f \Lambda \chi _n \Vert _{L^{\infty }({\mathbb {R}}^5\setminus {\mathbb {B}}_n^5)} \lesssim n^{-2}, \end{aligned}$$

we have $\Lambda f_n \in C^{\infty }_{c,\textrm{rad }}({\mathbb {R}}^5) \rightarrow \Lambda f$ in $L^{\infty }({\mathbb {R}}^5)$. By similar considerations as above one finds that $(\Lambda f_n)$ is Cauchy in $X^k$ and thus $\Lambda f_n \rightarrow \Lambda f$ in $X^k$ for $n \rightarrow \infty$. Now,

4.19 $$\begin{aligned} L f_n = \Delta f_n - \frac{1}{2} \Lambda f_n - f_n + 2 \Lambda (\phi f_n) + 12 \phi f_n. \end{aligned}$$

Arguments as above and the Banach algebra property of $X^k$ imply that $(L f_n)$ converges in $X^k$ to some limiting function $v \in X^k$. By Sobolev embedding, $L f_n \rightarrow v$ in $L^{\infty }({\mathbb {R}}^5)$ and by convergence of the individual terms, $v = \Delta f - \frac{1}{2} \Lambda f - f + 2 \Lambda (\phi f) + 12 \phi f.$ This shows in particular, that ${\mathcal {L}}_k$ acts as a classical differential operator on ${\mathcal {C}}$.

For the invocation of the Lumer–Phillips theorem, it is left to prove the density of the range of $\lambda _k - {\mathcal {L}}_k$ for some $\lambda _k > {\bar{\omega }}_k$. This crucial property is established by an ODE argument, the proof of which is rather technical and therefore provided in Appendix C. More precisely, let $f \in C^{\infty }_{c,\textrm{rad }}({\mathbb {R}}^5)$ such that $f = {{\tilde{f}}} (|\cdot |)$. By Lemma C.1, there exists $\lambda > {\bar{\omega }}_k$ such that the ODE

$$\begin{aligned}&{\tilde{u}}''(\rho ) + \left( \frac{4}{\rho } - \frac{1}{2} \rho + 2 \rho {{\tilde{\phi }}}(\rho ) \right) {\tilde{u}}'(\rho )\\&\quad + \left( 2\rho {{\tilde{\phi }}}'(\rho ) + 12 {{\tilde{\phi }}}(\rho ) - (\lambda _k + 1) \right) {\tilde{u}}(\rho ) = - {\tilde{f}}(\rho ), \end{aligned}$$

with $\phi = {{\tilde{\phi }}}(|\cdot |)$, has a solution ${{\tilde{u}}} \in C^1[0,\infty ) \cap C^{\infty }(0,\infty )$ satisfying ${{\tilde{u}}}'(0) = 0$ as well as ${{\tilde{u}}}^{(j)}(\rho ) = {\mathcal {O}}(\rho ^{-3-j})$ for $j \in {\mathbb {N}}_0$ as $\rho \rightarrow \infty$. By setting $u:= {{\tilde{u}}}(|\cdot |)$, we obtain a classical solution to the equation

4.20 $$\begin{aligned} (\lambda _k - L) u = f \end{aligned}$$

on ${\mathbb {R}}^5\setminus \{0\}$. Since *u* belongs to $H^1({\mathbb {R}}^5)$, it solves (4.20) weakly on ${\mathbb {R}}^5$, and by elliptic regularity we infer that $u \in C^{\infty }_{\textrm{rad}}({\mathbb {R}}^5)$. The decay of ${{\tilde{u}}}$ at infinity implies that $u \in {\mathcal {C}}$. Hence, $u \in {\mathcal {D}}( {\mathcal {L}}_k)$ which implies the claim.

An application of the Lumer–Phillips Theorem now proves that $({\mathcal {L}}_k, {\mathcal {D}}({\mathcal {L}}_k))$ generates a strongly continuous semigroup $(S_k(\tau ))_{\tau \geqq 0}$ on $X^k$. In view of the embedding $X^k \hookrightarrow {\mathcal {H}}$ and the fact that $C^{\infty }_{c,\textrm{rad}}({\mathbb {R}}^5)$ is a core for ${\mathcal {L}}$ and ${\mathcal {L}}_{k}$ for any *k*, an application of Lemma C.1 in [27] proves the claimed restriction properties. $\square$

In view of the restriction properties stated in Proposition 4.7, we can safely omit the index *k* in the notation of the semigroup.

Before turning to the proof of Theorem 4.1, we state a result for the free evolution, which follows in a straightforward manner analogous to the proof of Proposition 4.7, using in particular Equation (4.12) and setting $L'=0$ in the subsequent arguments.

#### Lemma 4.8

Let $k \in {\mathbb {N}}$, $k \geqq 3$. The operator $L_0: {\mathcal {D}}(L_0) \subset X^k \rightarrow X^k$ is closable and its closure $({\mathcal {L}}_{0,k}, {\mathcal {D}}({\mathcal {L}}_{0,k}))$ generates a strongly continuous semigroup on $X^k$, which coincides with the restriction of the $S_0(\tau )$ to $X^k$ for $\tau \geqq 0$. Furthermore,

$$\begin{aligned} \Vert S_0(\tau ) f \Vert _{X^k} \leqq e^{-\frac{\tau }{4}} \Vert f \Vert _{X^k} \end{aligned}$$

for all $f \in X^k$ and $\tau \geqq 0$.

### Proof of Theorem 4.1

First, we show that the operator ${\mathcal {P}}$ from (4.2) induces a non-orthogonal rank-one projection on $X^k$. To indicate the dependence on *k*, we write

4.21 $$\begin{aligned} {\mathcal {P}}_{X^{k}} f:= \langle f, g \rangle _{{\mathcal {H}}} ~ g \end{aligned}$$

for $f \in X^k$. By its decay properties, the function *g* is an element of ${\mathcal {C}} \subset X^k$ for any $k \in {\mathbb {N}}$. In view of the embedding $X^k \hookrightarrow {\mathcal {H}}$, the inner product makes sense for $f \in X^k$ and by definition ${\mathcal {P}}_{X^{k}}^2 = {\mathcal {P}}_{X^{k}}$. The fact that the projection commutes with $S_{k}(\tau )$ follows from the respective properties on ${\mathcal {H}}$.

Let $f \in C^\infty _{c,\textrm{rad}}({\mathbb {R}}^5)$. By Proposition 4.7, ${\tilde{f}}:=(1-{\mathcal {P}}_{X^{k}})f \in {\mathcal {C}} \subset {\mathcal {D}}({\mathcal {L}}_k)$. Using Equation (4.9), Lemmas 4.4 and 4.6, we infer that for $R \geqq 1$ sufficiently large,

$$\begin{aligned} \tfrac{1}{2} \tfrac{d}{d\tau } \Vert S(\tau ){\tilde{f}} \Vert ^2_{X^k}&= {\text {Re}}\langle \partial _{\tau } S(\tau ) {\tilde{f}}|S(\tau ) {\tilde{f}} \rangle _{X^k} = {\text {Re}}\langle {\mathcal {L}}_k S(\tau ) {\tilde{f}}|S(\tau ) {\tilde{f}}\rangle _{X^k} \\&\leqq \left( -\tfrac{1}{4} + \tfrac{C_k}{R^2}\right) \Vert S(\tau ) {\tilde{f}}\Vert ^2_{X^k} + C_{R,k} \sum _{j=0}^{k} \Vert S(\tau ){\tilde{f}} \Vert ^2_{{\mathcal {G}}((1- {\mathcal {L}})^{j/2})} \\&\leqq -\tfrac{1}{8} \Vert S(\tau ) {\tilde{f}}\Vert ^2_{X^k}+ C_{k} e^{-2 \omega _0 \tau } \sum _{j=0}^{k} \Vert {\tilde{f}} \Vert ^2_{{\mathcal {G}}((1- {\mathcal {L}})^{j/2})} \\&\leqq -\tfrac{1}{8} \Vert S(\tau ) {\tilde{f}}\Vert ^2_{X^k} + C_{k} e^{-2 \omega _0 \tau } \Vert {\tilde{f}} \Vert _{X^k}^2 \\ {}&\leqq - 2 c \Vert S(\tau ) {\tilde{f}}\Vert ^2_{X^k} + C_k e^{-4 c \tau } \Vert {\tilde{f}} \Vert ^2_{X^k}, \end{aligned}$$

for $$c = \frac{1}{2} \min \{\omega _0, \frac{1}{8} \}$$. Hence,

$$\begin{aligned} \tfrac{1}{2} \tfrac{d}{d\tau } \left[ e^{4 c \tau } \Vert S(\tau ) {\tilde{f}}\Vert ^2_{X^k} \right] \leqq C_k \Vert {\tilde{f}} \Vert ^2_{X^k}, \end{aligned}$$

and integration yields

$$\begin{aligned} \Vert S(\tau ) {\tilde{f}}\Vert ^2_{X^k} \leqq (1 + 2 C_k \tau ) e^{-4 c \tau } \Vert {\tilde{f}} \Vert ^2_{X^k} \lesssim e^{-2 \omega _k \tau } \Vert {\tilde{f}} \Vert ^2_{X^k}, \end{aligned}$$

for some suitably chosen $\omega _k > 0$ and all $\tau \geqq 0$. For $f \in X^k$, the same bound follows by density. Again, for simplicity, we write ${\mathcal {P}} f= {\mathcal {P}}_{X^k} f$ for $f \in X^k$.

## Nonlinear Estimates

Now we turn to the analysis of the full nonlinear Equation (2.8). In this section, we establish for the operator *N* a series of estimates which will be necessary later on for constructing solutions to (2.8). Recall that for $k \geqq 3$ the space $X^k$ embeds into $C^{k-3}_{\text {rad}}({\mathbb {R}}^5)$. Therefore, multiplication and taking derivatives of order at most $k-3$ is well defined for functions in $X^k$. With this in mind, we formulate and prove the following important lemma:

### Lemma 5.1

Let $k \in {\mathbb {N}}, k \geqq 4$. Given $f,g \in X^k$ we have that $\Lambda (fg) \in X^{k-1}$. Furthermore,

5.1 $$\begin{aligned} \Vert \Lambda (fg) \Vert _{X^{k-1}} \lesssim \Vert f\Vert _{X^{k}} \Vert g \Vert _{X^{k}}, \end{aligned}$$

for all $$f,g \in X^k$$.

### Proof

In this proof we crucially rely on a recently established inequality for the weighted $L^\infty$-norms of derivatives of radial Sobolev functions; see [27], Proposition B.1. For convenience, we copy here the version of this result in five dimensions. Namely, given $s \in (\frac{1}{2},\frac{5}{2})$ and $\alpha \in {\mathbb {N}}_0^5$, we have that

5.2 $$\begin{aligned} \Vert |\cdot |^{\frac{5}{2}-s} \partial ^\alpha u\Vert _{L^\infty ({\mathbb {R}}^5)} \lesssim \Vert u \Vert _{{\dot{H}}^{|\alpha |+s}({\mathbb {R}}^5)} \end{aligned}$$

for all $u \in C^\infty _{c,\text {rad}}({\mathbb {R}}^5)$. Now we turn to proving (5.1). Due to the $W^{1,\infty }$-embedding of $X^k$ for $k \geqq 4$ and the fact that $(f,g) \mapsto \Lambda (fg)$ is bilinear, it is enough to show (5.1) for $f,g \in C^\infty _{c,\text {rad}}({\mathbb {R}}^5)$. To estimate the ${\dot{H}}^{k-1}$ part, we do the following. If *k* is odd, then

$$\begin{aligned} \Vert D^{k-1} \Lambda (fg) \Vert _{L^2({\mathbb {R}}^5)}&= \Vert \Delta ^{\frac{k-1}{2}} \Lambda (fg) \Vert _{L^2({\mathbb {R}}^5)} \lesssim \sum _{|\alpha | + |\beta | = k} \Vert |\cdot | \partial ^\alpha f \, \partial ^\beta g \Vert _{L^2({\mathbb {R}}^5)} \\&\quad + \sum _{|\alpha | + |\beta | = k-1} \Vert \partial ^\alpha f \, \partial ^\beta g \Vert _{L^2({\mathbb {R}}^5)}\lesssim \Vert f \Vert _{X_k}\Vert g \Vert _{X_k}, \end{aligned}$$

where the last estimate follows from a combination of the $L^\infty$-embedding of $X_k$, Hardy’s inequality, and the inequality (5.2). To illustrate this, we estimate the first sum above. Without loss of generality we assume that $|\alpha | \leqq |\beta |$. We then separately treat the integrals corresponding to the unit ball and its complement. For the unit ball, we first assume that $\alpha =0$. Then

$$\begin{aligned} \Vert |\cdot | f \, \partial ^\beta g \Vert _{L^2({\mathbb {B}}^5)} \leqq \Vert f \Vert _{L^\infty ({\mathbb {R}}^5)} \Vert \partial ^\beta g\Vert _{L^2({\mathbb {R}}^5)} \lesssim \Vert f \Vert _{X_k}\Vert g \Vert _{X_k}, \end{aligned}$$

by the $L^\infty$-embedding. If $\alpha \ne 0$, we have that

$$\begin{aligned} \Vert |\cdot | \partial ^\alpha f \, \partial ^\beta g \Vert _{L^2({\mathbb {B}}^5)} \leqq \Vert |\cdot |^\frac{3}{2} \partial ^\alpha f \Vert _{L^\infty ({\mathbb {R}}^5)} \Vert |\cdot |^{-1} \partial ^\beta g\Vert _{L^2({\mathbb {R}}^5)} \lesssim \Vert f \Vert _{X_k}\Vert g \Vert _{X_k}, \end{aligned}$$

by (5.2) and Hardy’s inequality. For the complement of the unit ball, we have

$$\begin{aligned} \Vert |\cdot | \partial ^\alpha f \partial ^\beta g \Vert _{L^2({\mathbb {R}}^5\setminus {\mathbb {B}}^5)}&\leqq \Vert |\cdot |^{\frac{3}{2}}\partial ^\alpha f \Vert _{L^\infty ({\mathbb {R}}^5)} \Vert \partial ^\beta g \Vert _{L^2({\mathbb {R}}^5)} \lesssim \Vert f \Vert _{X_k}\Vert g \Vert _{X_k} \end{aligned}$$

by (5.2) only. The second sum is estimated similarly. If *k* is even, then we have that

$$\begin{aligned} \Vert D^{k-1} \Lambda (fg) \Vert _{L^2({\mathbb {R}}^5)} = \Vert \nabla \Delta ^{\frac{k-2}{2}}\Lambda (fg) \Vert _{L^2({\mathbb {R}}^5)}, \end{aligned}$$

and the desired estimate follows similarly to the previous case. The ${\dot{H}}^1$ part of the norm is treated in the same fashion. $\square$

Now we establish the crucial smoothing properties of $S_0(\tau )$.

### Proposition 5.2

Let $k \in {\mathbb {N}}, k \geqq 3$ and $l \in {\mathbb {N}}_0$. Then, given $f \in X^k$ the function

5.3 $$\begin{aligned} \tau \mapsto S_0(\tau )f \end{aligned}$$

maps $(0,\infty )$ continuously into $X^{k+l}$. Furthermore, denoting $\beta (\tau ):=\alpha (\tau )^{-\frac{1}{2}}$, where $\alpha$ is defined in Lemma 4.2, we have that

5.4 $$\begin{aligned} \Vert S_0(\tau ) f \Vert _{X^{k+l}}&\lesssim \beta (\tau )^{l} e^{-\frac{\tau }{4}}\Vert f \Vert _{X^k} \end{aligned}$$

for all $\tau >0$ and all $f \in X^k$.

### Proof

The proof follows from a straightforward computation on the Fourier side. Explicitly, by definition of $S_0(\tau )$, see Lemma 4.2, and scaling of Sobolev norms we get

$$\begin{aligned} \Vert S_0(\tau ) f \Vert _{\dot{H}^s({\mathbb {R}}^5)} = e^{\frac{\tau }{2}(\frac{1}{2}- s)} \Vert |\cdot |^{s} \hat{G}_{\tau } \hat{f} \Vert _{L^2({\mathbb {R}}^5)} \end{aligned}$$

for all $s \geqq 0$ and all $f \in C^{\infty }_{c,\textrm{rad}}({\mathbb {R}}^5)$. We infer that for all $f \in X^k$,

$$\begin{aligned} \Vert S_0(\tau ) f \Vert _{\dot{H}^{1}({\mathbb {R}}^5)} \lesssim e^{-\frac{\tau }{4}} \Vert f \Vert ^2_{\dot{H}^{1}({\mathbb {R}}^5)}, \end{aligned}$$

and

$$\begin{aligned} \Vert S_0(\tau ) f \Vert _{\dot{H}^{k+l}({\mathbb {R}}^5)} \lesssim \alpha (\tau )^{-\frac{l}{2}} e^{-\frac{\tau }{4}} \Vert f \Vert ^2_{\dot{H}^{k}({\mathbb {R}}^5)}, \end{aligned}$$

where we used that $\Vert |\cdot |^l \alpha (\tau )^{\frac{l}{2}} \hat{G}_{\tau }\Vert _{L^{\infty }({\mathbb {R}}^5)} \lesssim 1$ for all $\tau \geqq 0$. This implies (5.4). Continuity of the map $\tau \mapsto S_0(\tau )f: (0,\infty ) \rightarrow X^{k+l}$ follows from the continuity of the kernel maps $\hat{G}_{\tau }, |\cdot | \alpha (\tau )^{\frac{1}{2}} \hat{G}_{\tau }: (0,\infty ) \rightarrow L^{\infty }({\mathbb {R}}^5)$. $\square$

Now we propagate the smoothing estimates of $S_0(\tau )$ to $S(\tau )$. We take a perturbative approach, and for that we need the following result:

### Lemma 5.3

Let $f \in C^\infty _{c,rad }({\mathbb {R}}^5)$, $k \geqq 3$, and $\tau \geqq 0$. Then the following relations hold in $X^k$:

5.5 $$\begin{aligned} S(\tau )f= & {} S_0(\tau )f + \int _{0}^{\tau }S(\tau -s)L'S_0(s)f \textrm{d}s, \end{aligned}$$

5.6 $$\begin{aligned} S(\tau )f= & {} S_0(\tau )f + \int _{0}^{\tau }S_0(\tau -s)L'S(s)f \textrm{d}s. \end{aligned}$$

### Proof

Define $\xi _f:[0,\tau ] \rightarrow X^k$ by $s \mapsto S(\tau -s)S_0(s)f$. We prove that $\xi _f$ is continuously differentiable. More precisely, we show that

5.7 $$\begin{aligned} \xi _f'(s)= & {} -S(\tau -s)LS_0(s)f+S(\tau -s)L_0 S_0(s)f\nonumber \\= & {} - S(\tau -s) L' S_0(s)f, \end{aligned}$$

which is a continuous function from $[0,\tau ]$ into $X^k$. To show this, we first write

$$\begin{aligned} \frac{\xi _f(s+h)-\xi _f(s)}{h}&= \frac{S(\tau -s-h)-S(\tau -s)}{h}S_0(s)f \\&\quad + S(\tau -s-h)\frac{S_0(s+h)f-S_0(s)f}{h}, \end{aligned}$$

and then by letting $h \rightarrow 0$ we get (5.7). For the first term above, this follows from the fact that $S_0(s)f \in {\mathcal {C}} \subset {\mathcal {D}}({\mathcal {L}}_k)$ and that ${\mathcal {L}}_kf=Lf$ for $f \in {\mathcal {C}}.$ The conclusion for the second term follows by similar reasoning for $S_0(\tau )$, together with the strong continuity of $S(\tau )$ in $X^k$. Now, continuity of $\xi '_f$ follows from the continuity of the map

5.8 $$\begin{aligned} s \mapsto L' S_0(s)f:[0,\tau ] \rightarrow X^k \end{aligned}$$

and the strong continuity of $S(\tau )$ in $X^k$. We note that, according to the definition of $L'$, the continuity of (5.8) follows from the strong continuity of $S_0(\tau )$ on $X^{k+1}$ and the estimate (5.1). Finally, by integrating (5.7), we get (5.5). To prove (5.6), we do the analogous thing. Namely, we consider the function

$$\begin{aligned} s \mapsto \eta _f(s):=S_0(\tau -s)S(s)f: [0,\tau ] \rightarrow X^k, \end{aligned}$$

which is also continuously differentiable, with

$$\begin{aligned} \eta '_f(s)= S_0(\tau -s)LS(s)f - S_0(\tau -s)L_0S(s)f = S_0(\tau -s) L' S(s)f. \end{aligned}$$

To establish differentiability, it is important to note that according to the definition of the operator domain, by Lemma 5.1 we have that that ${\mathcal {D}}({\mathcal {L}}_{k+1}) \subset {\mathcal {D}}({\mathcal {L}}_{0,k})$, and therefore $S(s)f \in {\mathcal {D}}({\mathcal {L}}_{0,k})$ for every $k \geqq 3$. Continuity of $\eta '_f$, similarly to above, follows from the continuity of $s \mapsto L' S(s)f:[0,\tau ] \rightarrow X^k$ and the strong continuity of $S_0(\tau )$ in $X^k$. $\square$

Recall the operator *N* from (3.4). According to Lemma 5.1 we have that $N: X^{k} \rightarrow X^{k-1}$ for $k \geqq 4$. Also, recall the projection operator ${\mathcal {P}}={\mathcal {P}}_{X^k}$ from (4.21). Now we prove the central result of this section.

### Proposition 5.4

Let $k \geqq 3$. If $f \in X^k$ then

5.9 $$\begin{aligned} \tau \mapsto S(\tau )f:(0,\infty ) \rightarrow X^{k+1} \end{aligned}$$

is a continuous map. Furthermore, there exists $\tilde{\omega }_k >0$ such that

5.10 $$\begin{aligned} \Vert S(\tau )f \Vert _{X^{k+1}}&\lesssim \beta (\tau )e^{\tilde{\omega }_k\tau }\Vert f \Vert _{X^k}, \end{aligned}$$

5.11 $$\begin{aligned} \Vert S(\tau )(1-{\mathcal {P}})f \Vert _{X^{k+1}}&\lesssim \beta (\tau )e^{-\omega _{k+1} \tau }\Vert (1-{\mathcal {P}})f \Vert _{X^k}, \end{aligned}$$

for all $\tau > 0$ and all $f \in X^k$, with $\omega _{k+1} > 0$ from Theorem 4.1.

### Proof

The proof we give here is based exclusively on semigroup methods. In the Appendix 7 we provide a more standard proof by energy methods. We first prove (5.10). To this end, we use (5.6), (5.4), and (5.1) to obtain

$$\begin{aligned} \Vert S(\tau )f \Vert _{X^{k+1}}&\lesssim \beta (\tau )e^{-\frac{\tau }{4}}\Vert f \Vert _{X^{k}} + \int _{0}^{\tau } \beta (\tau -s)e^{-\frac{(\tau -s)}{4}} \Vert S(s)f\Vert _{X^{k+1}} ds \\&\lesssim \beta (\tau )e^{-\frac{\tau }{4}}\Vert f\Vert _{X^k} + e^{-\frac{\tau }{4}} \int _{0}^{\tau } \beta (\tau -s) e^{\frac{s}{4}} \Vert S(s)f \Vert _{X^{k+1}} ds, \end{aligned}$$

for all $\tau >0$ and $f \in C^\infty _{c,\text {rad}}({\mathbb {R}}^5)$. Now, a generalization of Gronwall’s lemma to weakly singular kernels (see, e.g., Henry [31], p. 188, Theorem 7.1.1) yields (5.10) for all $\tau \in (0,2)$. To treat higher values of $\tau$ we first note that, according to (5.6), for $\tau \geqq 2$ we have that

$$\begin{aligned} S(\tau )f&= S_0(\tau )f + \int _{0}^{\tau -1}S(\tau -s)L'S_0(s)fds + \int _{\tau -1}^{\tau }S(\tau -s)L'S_0(s)fds \\&= S_0(\tau )f + \int _{0}^{\tau -1}S(1)S(\tau -1-s)L'S_0(s)fds + \int _{\tau -1}^{\tau }S(\tau -s)L'S_0(s)fds. \end{aligned}$$

From here, by the previous step (for small $\tau$), Theorem 4.1, Lemma 5.1 and smoothing of $S_0(\tau )$, we get

$$\begin{aligned} \Vert S(\tau )f \Vert _{X^{k+1}} \lesssim&~ \beta (\tau )e^{-\frac{\tau }{4}}\Vert f \Vert _{X^k} + \int _{0}^{\tau -1}e^{(\tau -1-s)}\beta (s)\Vert f\Vert _{X^k}ds \\&+ \int _{\tau -1}^{\tau }e^{ (\tau -s)}\beta (s)^2\Vert f \Vert _{X^k} ds \end{aligned}$$

wherefrom follows the estimate (5.10) for all $\tau \geqq 2$.

Now we use (5.10), (5.5) and the decay of $S(\tau )$ on the orthogonal space to establish (5.11). We separately treat small and large values of $\tau$. First note that from (5.10) it follows that (5.11) holds for all $\tau \in (0,2)$. Now assume $\tau \geqq 2$. Then, we can write

$$\begin{aligned} S(\tau )f&= S_0(\tau )f + \int _{0}^{1}S(\tau -s)L'S_0(s)fds + \int _{1}^{\tau }S(\tau -s)L'S_0(s)fds \\&= S_0(\tau )f + S(\tau -1)\int _{0}^{1}S(1-s)L'S_0(s)fds + \int _{1}^{\tau }S(\tau -s)L'S_0(s)fds. \end{aligned}$$

Now, denote $\tilde{f}:=(1-{\mathcal {P}})f$. Then, according to Theorem 4.1, estimate (5.10), and smoothing of $S_0(\tau )$ we have that

$$\begin{aligned} \Vert S(\tau )\tilde{f} \Vert _{X^{k+1}} =&~\Vert (1- {\mathcal {P}}) S(\tau )\tilde{f} \Vert _{X^{k+1}} \\ \lesssim&~\beta (\tau )e^{-\frac{\tau }{4}}\Vert \tilde{f} \Vert _{X^{k}} + e^{-\omega _{k+1}\tau }\int _{0}^{1}\Vert S(1-s)L'S_0(s)\tilde{f} \Vert _{X^{k+1}}ds \\&+ \int _{1}^{\tau } \Vert S(\tau -s)(1-{\mathcal {P}})L' S_0(s)\tilde{f} \Vert _{X^{k+1}}ds \\ \lesssim&~ \beta (\tau )e^{-\frac{\tau }{4}}\Vert \tilde{f} \Vert _{X^{k}} + e^{-\omega _{k+1}\tau }\int _{0}^{1}\beta (1-s) e^{\tilde{\omega }_{k}(1-s)} \Vert L'S_0(s)\tilde{f} \Vert _{X^{k}}ds \\ {}&+ \int _{1}^{\tau } e^{-\omega _{k+1}(\tau -s)}\Vert L' S_0(s)\tilde{f} \Vert _{X^{k+1}}ds \\ \lesssim&~ \beta (\tau )e^{-\frac{\tau }{4}}\Vert \tilde{f} \Vert _{X^{k}} + e^{-\omega _{k+1}\tau }\int _{0}^{1}\beta (1-s)\beta (s) \Vert \tilde{f} \Vert _{X^k} \\ {}&+ e^{-\omega _{k+1}\tau } \int _{1}^{\tau } e^{\omega _{k+1} s}\beta (s)^2e^{-\frac{1}{4}s}\Vert \tilde{f} \Vert _{X^{k}}ds \\ \lesssim&~ \beta (\tau )e^{-\frac{\tau }{4}}\Vert \tilde{f} \Vert _{X^{k}} + e^{-\omega _{k+1}\tau } \Vert \tilde{f} \Vert _{X^k} + e^{-\omega _{k+1}\tau }\Vert \tilde{f} \Vert _{X^k}, \end{aligned}$$

for all $\tau \geqq 2$. From here, estimate (5.11) follows. The continuity of the map $S(\tau )f: (0,\infty ) \rightarrow X^{k+1}$ for $f \in X^{k}$ follows from (5.10) and the strong continuity of $S(\tau )$ in $X^k$. $\square$

As the last result of this section, we prove the local Lipschitz continuity in $X^4$ of the composition of ${\mathcal {P}}$ and *N*.

### Lemma 5.5

We have that

5.12 $$\begin{aligned} \Vert {\mathcal {P}}N(f)- {\mathcal {P}}N(g) \Vert _{X^4} \lesssim (\Vert f \Vert _{X^4} + \Vert g \Vert _{X^4}) \Vert f-g \Vert _{X^4}, \end{aligned}$$

for all $$f,g \in X^4$$.

### Proof

By definition, for $u,v \in X^4$ we have

$$\begin{aligned} {\mathcal {P}} N(uv) = \langle N(uv),g\rangle _{{\mathcal {H}}} \, g. \end{aligned}$$

Therefore, by Cauchy-Schwarz, the embedding $X^3 \hookrightarrow {\mathcal {H}}$, and Lemma 5.1, we get that

$$\begin{aligned} \Vert {\mathcal {P}}N(uv) \Vert _{X^4} \leqq |\langle N(uv),g\rangle _{{\mathcal {H}}}| \Vert g \Vert _{X^4} \lesssim \Vert N(uv) \Vert _{X^3} \lesssim \Vert u \Vert _{X^4}\Vert v \Vert _{X^4}, \end{aligned}$$

for all $u,v \in X^4$. The estimate (5.12) then follows by letting $u=f+g$ and $v=f-g$. $\square$

## Construction of Strong Solutions

For simplicity, from now on we will drop the subscript in $\Vert \cdot \Vert _{X^4}$, and assume that an unspecified norm corresponds to $X^4$. With the linear theory and the nonlinear estimates from the previous section at hand, we turn to constructing solutions to (2.8). For convenience, we copy here the underlying Cauchy problem

6.1 $$\begin{aligned} {\left\{ \begin{array}{ll} ~\partial _\tau \psi (\tau ) = {\mathcal {L}}\psi (\tau ) + N(\psi (\tau )),\\ ~\psi (0)= U( v, T). \end{array}\right. } \end{aligned}$$

To solve (6.1), we utilize the standard techniques from dynamical systems theory. First, we use the fact that ${\mathcal {L}}$ generates the semigroup $S(\tau )$, to rewrite (6.1) into integral form

6.2 $$\begin{aligned} \psi (\tau )= S(\tau ) U(v,T) + \int _{0}^{\tau } S(\tau -s) N(\psi (s))\textrm{d}s. \end{aligned}$$

Then, as $S(\tau )$ decays exponentially on the stable subspace, we employ a fixed point argument to show existence of global solutions for small initial data. Obstruction to this is, of course, the presence of the linear instability $\lambda =1$. Nevertheless, as this eigenvalue is an artifact of the time translation symmetry, we use a Lyapunov-Perron type argument to suppress it by appropriately choosing the blowup time. Before stating the first result, we make some technical preparations. First, we introduce the Banach space

$$\begin{aligned} {\mathcal {X}}:= \{ \psi \in C([0,\infty ), X^4): \Vert \psi \Vert _{{\mathcal {X}}}:= \sup _{\tau >0}e^{\omega \tau }\Vert \psi (\tau )\Vert < \infty \}, \end{aligned}$$

where $\omega _4$ is from Proposition 5.4. Then, we denote

$$\begin{aligned} {\mathcal {X}}_\delta := \{ \psi \in {\mathcal {X}}: \Vert \psi \Vert _{{\mathcal {X}}} \leqq \delta \}. \end{aligned}$$

Now, we define a correction function $C: X^4 \times {\mathcal {X}} \rightarrow X^4$ by

6.3 $$\begin{aligned} C( u, \psi ):= {\mathcal {P}} \left( u + \int _{0}^{\infty }e^{- s} N(\psi (s))\textrm{d}s \right) , \end{aligned}$$

and a map $K_{u}: {\mathcal {X}} \rightarrow C([0,\infty ),X^4)$ by

$$\begin{aligned} K_{ u}( \psi )(\tau ):= S(\tau )\big ( u - C( u, \psi ) \big ) + \int _{0}^{\tau } S(\tau -s) N(\psi (s))\textrm{d}s. \end{aligned}$$

The fact that $K_u(\psi )(\tau )$ is a well-defined element of $X^4$ for every $\tau \geqq 0$, follows from Theorem 4.1, Proposition 5.4 and the integrability of $\beta$. Similarly, the continuity of $K_u(\psi ):[0,\infty ) \rightarrow X^4$ follows.

### Proposition 6.1

For all sufficiently small $\delta >0$ and all sufficiently large $C > 0$ the following holds. If $u \in {\mathcal {B}}_{\delta /C}$ then there exits a unique $\psi = \psi ({u}) \in {\mathcal {X}}_\delta$ for which

6.4 $$\begin{aligned} \psi = { K}_{u}( \psi ). \end{aligned}$$

Furthermore, the map ${u} \mapsto \psi ({u}): {\mathcal {B}}_{\delta /C} \rightarrow {\mathcal {X}}$ is Lipschitz continuous.

### Proof

To utilize the decay of $$ S(\tau )$$ on the stable subspace, we write $$ K_{u}$$ in the following way:

$$\begin{aligned} K_{u}( \psi )(\tau )&= S(\tau )(1- {\mathcal {P}}) u + \int _{0}^{\tau } S(\tau -s) (1 - {\mathcal {P}}) N(\psi (s))\textrm{d}s \\&\quad - \int _{\tau }^{\infty } e^{\tau - s} {\mathcal {P}} N(\psi (s))\textrm{d}s. \end{aligned}$$

Then, according to Proposition 5.4 we get that if $\psi (s) \in {\mathcal {B}}_\delta$ for all $s \geqq 0$ then

$$\begin{aligned} \Vert K_{ u}( \psi )(\tau ) \Vert \lesssim e^{-\omega \tau }\Vert u \Vert + e^{-\omega \tau } \int _{0}^{\tau } \beta (\tau - s) e^{\omega s}\Vert \psi (s) \Vert ^2ds + e^{\tau }\int _{\tau }^{\infty } e^{-s} \Vert \psi (s) \Vert ^2 \textrm{d}s. \end{aligned}$$

Furthermore, since $\beta$ is integrable, if $u \in {\mathcal {B}}_{\delta /C}$ and $\psi \in {\mathcal {X}}_{\delta }$ then the above estimate implies the bound

$$\begin{aligned} e^{\omega \tau }\Vert K_{ u}( \psi )(\tau ) \Vert \lesssim \tfrac{\delta }{C} + \delta ^2 + \delta ^2 e^{-\omega \tau }. \end{aligned}$$

Also, we similarly get that

$$\begin{aligned} e^{\omega \tau }\Vert K_{ u}( \psi )(\tau ) - K_{ u}( \varphi )(\tau ) \Vert \lesssim (\delta + \delta e^{-\omega \tau })\Vert \psi - \varphi \Vert _{{\mathcal {X}}} \end{aligned}$$

for all $$\psi ,\varphi \in {\mathcal {X}}_\delta $$. Now, the last two displayed equations imply that for all small enough $$\delta $$ and for all large enough *C*, given $$ u \in {\mathcal {B}}_{\delta /C}$$ the operator $$ K_{ u}$$ is contractive on $${\mathcal {X}}_\delta $$, with the contraction constant $$\frac{1}{2}$$. Consequently, the existence and uniqueness of solutions to (6.4) follows from the Banach fixed point theorem. To show continuity of the map $$ u \mapsto \psi ( u) $$ we utilize the contractivity of $$ K_{ u}$$. Namely, we have the estimate

$$\begin{aligned} \Vert \psi ( u)(\tau ) - \psi ( v)(\tau ) \Vert&= \Vert K_{ u}( \psi ( u))(\tau ) - K_{ v}( \psi ( v))(\tau )\Vert \\&\leqq \Vert K_{ u}( \psi ( u))(\tau ) - K_{ v}( \psi ( u))(\tau )\Vert \\&\quad + \Vert K_{ v}( \psi ( u))(\tau ) - K_{ v} (\psi ( v))(\tau )\Vert \\&\leqq \Vert S(\tau )(1- {\mathcal {P}})( u- v) \Vert + \tfrac{1}{2}\Vert \psi ( u)(\tau ) - \psi ( v)(\tau ) \Vert \\&\leqq Ce^{-\omega \tau } \Vert u - v \Vert + \tfrac{1}{2}\Vert \psi ( u)(\tau ) - \psi ( v)(\tau ) \Vert , \end{aligned}$$

wherefrom the Lipschitz continuity follows. $\square$

### Lemma 6.2

Recall the initial data operator *U* from (2.10). For $\delta \in (0,\frac{1}{2}]$ and ${ v} \in X^4$ the map

$$\begin{aligned} T \mapsto { U}({ v},T):[1-\delta ,1+\delta ] \rightarrow X^4 \end{aligned}$$

is continuous. In addition, we have that

6.5 $$\begin{aligned} \Vert { U}({ v},T) \Vert \lesssim \Vert { v} \Vert + |T-1| \end{aligned}$$

for all ${ v} \in X^4$ and all $T \in [\frac{1}{2},\frac{3}{2}]$.

### Proof

Fix $\delta \in (0,\frac{1}{2}]$ and $v \in X^4$. Then for $T,S \in [1-\delta ,1+\delta ]$ we have that

6.6 $$\begin{aligned} U(v,T) - U(v,S) = (T-S)w_0(\sqrt{T}\cdot ) + S\big ( w_0(\sqrt{T}\cdot ) - w_0(\sqrt{S}\cdot ) \big ). \end{aligned}$$

Let $\varepsilon >0$. By density, we know that there exists ${\tilde{w}}_0 \in C^\infty _{c,\text {rad}}({\mathbb {R}}^5)$ for which $\Vert w_0 - {\tilde{w}}_0 \Vert < \varepsilon$. Now, by writing

$$\begin{aligned} w_0(\sqrt{T}\cdot ) - w_0(\sqrt{S}\cdot ) = \big (&w_0(\sqrt{T}\cdot ) - {\tilde{w}}_0(\sqrt{T}\cdot ) \big ) \\ {}&+ \big ( {\tilde{w}}_0(\sqrt{T}\cdot ) - {\tilde{w}}_0(\sqrt{S}\cdot ) \big ) + \big ( {\tilde{w}}_0(\sqrt{S}\cdot ) - w_0(\sqrt{S}\cdot ) \big ) \end{aligned}$$

and using the fact that $\lim _{S \rightarrow T} \Vert {\tilde{w}}_0(\sqrt{T}\cdot ) - {\tilde{w}}_0(\sqrt{S}\cdot ) \Vert =0,$ from (6.6) we see that

$$\begin{aligned} \lim _{S \rightarrow T} \Vert U( v,T) - U( v,S) \Vert \lesssim \varepsilon . \end{aligned}$$

Then, continuity follows by letting $\varepsilon \rightarrow 0$. For the second part of the lemma, we write *U*(*v*, *T*) in the following way

6.7 $$\begin{aligned} U( v,T) = Tv(\sqrt{T}\cdot ) + T \phi (\sqrt{T}\cdot )-\phi \end{aligned}$$

From here, the estimate (6.5) follows. $\square$

Finally, by using the results above, we prove that given initial datum *v* that is small in $X^4$, there exists a time *T* and an exponentially decaying solution $\psi \in C([0,\infty ),X^4)$ to (6.2).

### Theorem 6.3

There exist $\delta ,N>0$ such that the following holds. If

6.8 $$\begin{aligned} { v} \in X^4, \quad v \text { is real-valued}, \quad \text {and} \quad \Vert { v} \Vert \leqq \tfrac{\delta }{N^2}, \end{aligned}$$

then there exist $T \in [1-\frac{\delta }{N}, 1+\frac{\delta }{N}]$ and $\psi \in {\mathcal {X}}_{\delta }$ such that (6.2) holds for all $\tau \geqq 0.$

### Proof

Lemma 6.2 and Proposition 6.1 imply that for all small enough $\delta$ and all large enough *N* we have that if *v* satisfies (6.8) and $T \in [1-\frac{\delta }{N}, 1+\frac{\delta }{N}]$ then there is a unique $\psi =\psi ({ v},T) \in {\mathcal {X}}_\delta$ that solves

6.9 $$\begin{aligned} \psi (\tau ) = S(\tau )\big ( U( v,T) - C( U( v,T), \psi ) \big ) + \int _{0}^{\tau } S(\tau -s) N\big (\psi (s)\big )\textrm{d}s. \end{aligned}$$

We remark that $\psi (\tau )$ is real-valued for all $\tau \geqq 0$, since the set of real-valued functions in $X^4$ is invariant under the action of both $S(\tau )$ and ${\mathcal {P}}$. Now, to construct solutions to (6.2), we prove that there is a choice of $\delta$ and *N* such that for any *v* that satisfies (6.8) there is $T=T( v) \in [1-\frac{\delta }{N}, 1+\frac{\delta }{N}]$ for which the correction term in (6.9) vanishes. As *C* takes values in ${\text {rg}}{\mathcal {P}} = \langle g \rangle$, it is enough to show existence of *T* for which

6.10 $$\begin{aligned} \big \langle C( U( v,T), \psi ({ v},T)), g\big \rangle _{X^4}=0. \end{aligned}$$

We therefore consider the real function $T \mapsto \langle C( U( v,T), \psi ({ v},T)), g \rangle _{X^4}$ and employ the intermediate value theorem to prove that it vanishes on $[1-\frac{\delta }{N}, 1+\frac{\delta }{N}]$. The central observation to this end is that, according to (4.1), we have

$$\begin{aligned} \partial _T \, T \phi (\sqrt{T}\cdot ) \big |_{T=1} = c g, \end{aligned}$$

for some $c>0$. Based on this, by Taylor’s formula, from (6.7) we get that

$$\begin{aligned} \big \langle {\mathcal {P}} U( v,T), g \big \rangle _{X^4}= c \Vert g\Vert ^2 \,(T-1)+R_1( v,T), \end{aligned}$$

where $R_1( v,T)$ is continuous in *T* and $R_1( v,T) \lesssim \delta /N^2$.

Furthermore, based on the definition of the correction function *C*, we similarly conclude that

$$\begin{aligned} \big \langle C( U( v,T), \psi ({ v},T)), g \big \rangle _{X^4}= c \Vert g\Vert ^2 \,(T-1) + R_2( v, T), \end{aligned}$$

where $T \mapsto R_2( v,T)$ is a continuous, real-valued function on $[1-\frac{\delta }{N}, 1+\frac{\delta }{N}]$, for which $R_2( v,T) \lesssim \delta /N^2 + \delta ^2.$ Therefore, there is a choice of sufficiently large *N* and sufficiently small $\delta$ such that $|R_2( v,T)| \leqq c \Vert g \Vert ^2 \frac{\delta }{N}$. Based on this, we get that (6.10) is equivalent to

6.11 $$\begin{aligned} T=F(T) \end{aligned}$$

for some function *F* which maps the interval $[1-\frac{\delta }{N}, 1+\frac{\delta }{N}]$ continuously into itself. Consequently, by the intermediate value theorem we infer the existence of $T \in [1-\frac{\delta }{N}, 1+\frac{\delta }{N}]$ for which (6.11), and therefore (6.10), holds. The claim of the theorem follows. $\square$

## Upgrade to Classical Solutions

In this section we show that if the initial datum *v* is smooth and rapidly decaying, then the corresponding strong solution to (6.2) is in fact smooth, and satisfies (6.1) classically. To accomplish this, we first use abstract results of semigroup theory to upgrade strong solutions to classical ones in the semigroup sense. Then we use repeated differentiation together with Schwarz’s theorem on mixed partials to upgrade these to smooth solutions that solve (6.1) classically.

### Proposition 7.1

If *v* from Theorem 6.3 belongs to the radial Schwartz class ${\mathcal {S}}_{rad }({\mathbb {R}}^5)$, then the function $\Psi (\tau ,\xi ):=\psi (\tau )(\xi )$ belongs to $C^\infty ([0,\infty )\times {\mathbb {R}}^5)$ and satisfies

7.1 $$\begin{aligned} \partial _\tau \Psi (\tau ,\cdot ) = L \Psi (\tau ,\cdot ) + N(\Psi (\tau ,\cdot )) \end{aligned}$$

in the classical sense.

### Proof

Instead of $\psi (\tau )$ solving (6.2), we analyze the corresponding strong solution to (2.6). More precisely, by means of relation (5.6), we get that $\tau \mapsto \varphi (\tau ) := \phi + \psi (\tau )$ belongs to $C([0,\infty ),X^4)$ and satisfies

7.2 $$\begin{aligned} \varphi (\tau )= S_0(\tau ) U_0(T) + \int _{0}^{\tau } S_0(\tau -s) N(\varphi (s))ds, \end{aligned}$$

where $U_0(T)=\phi +U(v,T)$. First, we show that $\varphi (\tau )$ belongs to $C^{\infty }({\mathbb {R}}^5)$ for all $\tau \geqq 0$. To this end, we use the following two estimates

7.3 $$\begin{aligned} \Vert \Lambda (fg) \Vert _{X^{\frac{k}{2}}} \lesssim \Vert f \Vert _{X^{\frac{k}{2}+1}} \Vert g \Vert _{X^{\frac{k}{2}+1}} \end{aligned}$$

for $$k \geqq 6$$, and

7.4 $$\begin{aligned} \Vert S_0(\tau )f \Vert _{X^{\frac{k}{2}}} \lesssim \beta (\tau )^\frac{l}{2}\Vert f \Vert _{X^{\frac{k-l}{2}}} \end{aligned}$$

for $k > 2 + l$ and $l \geqq 0$. To remove any possible ambiguity in notation, by $X^s$ for a non-integer *s*, we denote, analogous to the integer exponent case, the closure of the test space $C^\infty _{\text {rad}}({\mathbb {R}}^5)$ under the norm $\Vert \cdot \Vert ^2_{X^s} = \Vert \cdot \Vert ^2_{\dot{H}^1({\mathbb {R}}^5)} + \Vert \cdot \Vert ^2_{\dot{H}^s({\mathbb {R}}^5)}$. The estimates (7.3) and (7.4) are proved by using standard arguments from interpolation theory (see, e.g., Bergh-Löfström [2], Section 6.4, as well as Theorem 4.4.1, p. 96) together with (5.1) and Proposition 5.2. Now, from (7.2) and the above estimates, we have

$$\begin{aligned} \Vert \varphi (\tau ) \Vert _{X^{\frac{9}{2}}}&\lesssim \Vert U_0(T) \Vert _{X^{\frac{9}{2}}} + \int _{0}^{\tau }\beta (\tau -s)^{\frac{3}{2}} \Vert \varphi (s)\Vert ^2_{X^{4}}ds, \end{aligned}$$

which implies that $\varphi (\tau ) \in X^{\frac{9}{2}}$ for all $\tau \geqq 0$. Then, inductively we get that $\varphi (\tau ) \in X^\frac{k}{2}$ for all $k \geqq 9$ and $\tau \geqq 0$. Then, by the embedding $X^k \hookrightarrow C^{k-3}_{\text {rad}}({\mathbb {R}}^5)$ we conclude that $\psi (\tau ) \in C^\infty ({\mathbb {R}}^5)$ for all $\tau \geqq 0$. To establish regularity in $\tau$, we do the following. First, since $\varphi$ is a locally bounded curve in $X^5$ we use Gronwall’s inequality (see, e.g., Cazenave-Haraux [11], p. 55, Lemma 4.2.1) to show from (7.2) that $\varphi :[0,{\mathcal {T}}] \rightarrow X^4$ is Lipschitz continuous for every ${\mathcal {T}}>0$. Consequently, according to (5.1) we have that $\tau \mapsto N(\psi (\tau )):[0,{\mathcal {T}}] \rightarrow X^3$ is Lipschitz continuous for every ${\mathcal {T}} >0$. This, together with the fact that $U_0(T) \in {\mathcal {D}}( {\mathcal {L}}_0)$ implies that $\varphi \in C^1([0,\infty ),X^3)$, and therefore $\psi (\tau )=\varphi (\tau ) - \phi$ satisfies (6.1) in $X^3$ in the operator sense (see, e.g., [11], p. 51, Proposition 4.1.6, (ii)). Furthermore, as $X^3$ is continuously embedded in $L^\infty ({\mathbb {R}}^5)$ the $\tau$-derivative holds pointwise. Consequently, by (a strong version of) the Schwarz theorem (see, e.g., Rudin [49], p. 235, Theorem 9.41), we conclude that mixed derivatives of all orders in $\tau$ and $\xi$ exist, and we thereby infer smoothness of $(\tau ,\xi ) \mapsto \psi (\tau )(\xi )$ and the fact that it satisfies (7.1) classically. $\square$

### Proof of Theorem 1.1

Due to Lemma B.1 we can choose $\varepsilon >0$ small enough such that

$$\begin{aligned} \Vert \varphi _0 \Vert _{H^{3}({\mathbb {R}}^3)}< \varepsilon \quad \text {implies} \quad \Vert v \Vert _{{\dot{H}}^1 \cap {\dot{H}}^{4}({\mathbb {R}}^5)} < \frac{\delta }{N^2}, \end{aligned}$$

for $\delta ,N$ from Theorem 6.3. Then, according to Theorem 6.3 there exists a solution $\psi \in C([0,\infty ),X^4)$ to (6.2), for which

7.5 $$\begin{aligned} \Vert \psi (\tau )\Vert _{X^4} \leqq \delta e^{-\omega \tau }. \end{aligned}$$

Now, since, by assumption, *v* belongs to ${\mathcal {S}}_{\text {rad}}({\mathbb {R}}^5)$, Proposition 7.1 implies that $\Psi (\tau ,\xi ) = \phi (\xi ) + \psi (\tau )(\xi )$ is smooth and solves (2.6) classically. Therefore,

$$\begin{aligned} w(t,x)=\frac{1}{T-t}{\Psi }\left( \log \left( \frac{T}{T-t}\right) ,\frac{x}{T-t}\right) \end{aligned}$$

belongs to $C^\infty ([0,T)\times {\mathbb {R}}^5)$ and solves the system (2.1) on $[0,T)\times {\mathbb {R}}^5$ classically. This then yields a smooth solution to (1.1)

$$\begin{aligned} u(t,x)=\frac{1}{T-t}\left[ \Phi \left( \frac{x}{\sqrt{T-t}}\right) + \varphi \left( t, \frac{x}{\sqrt{T-t}}\right) \right] , \end{aligned}$$

where, according to (B.1) and (7.5) we have

$$\begin{aligned} \Vert \varphi (t,\cdot ) \Vert _{H^3({\mathbb {R}}^3)}&\simeq \Vert \varphi (t,\cdot ) \Vert _{L^2 \cap {\dot{H}}^3({\mathbb {R}}^3)} \\ {}&\simeq \Vert \psi (-\log (T-t)-\log T) \Vert _{{\dot{H}}^1 \cap {\dot{H}}^4({\mathbb {R}}^5)} \lesssim (T-t)^\omega , \end{aligned}$$

as $$t \rightarrow T^-$$. $$\square $$

## Data Availability

No datasets were generated or analyzed during the current study.

## Appendix A. Estimates of Local Sobolev Norms

### Lemma A.1

Let $k \in {\mathbb {N}}$ and $R>0.$ Then

A.1 $$\begin{aligned} \Vert \partial ^\alpha u \Vert _{L^2({\mathbb {B}}^5_R)} \lesssim \sum _{j=0}^{k} \Vert D^j u \Vert _{L^2({\mathbb {B}}^5_R)} \end{aligned}$$

for all $u \in C^\infty _{rad }({\mathbb {B}}^5_R)$ and all $\alpha \in {\mathbb {N}}_0^5$ with $|\alpha |=k$.

### Proof

We prove the claim for $R=1$ as the general case follows by scaling. Let $\chi : {\mathbb {R}}^5 \rightarrow [0,1]$ be a smooth radial function such that $\chi (x) =0$ for $|x|\leqq \frac{5}{4}$ and $\chi (x) = 0$ for $|x| \geqq \frac{3}{2}$. We then define for $u={\tilde{u}}(|\cdot |) \in C_{\text {rad}}^k({\mathbb {B}}^5)$ the extension operator

$$\begin{aligned} {\tilde{E}}u:= {\left\{ \begin{array}{ll} u(x), \quad &{} x \in {\mathbb {B}}^5, \\ -{\tilde{u}}(2-|x|) + 2 \displaystyle {\sum _{j=0}^{\frac{k-1}{2}}} \dfrac{ {\tilde{u}}^{(2j)}(1)}{(2j)!}(|x|-1)^{2j}, &{} x \in {\mathbb {B}}^5_2 \setminus {\mathbb {B}}^5, ~k \text { odd},\\ {\tilde{u}}(2-|x|) + 2 \displaystyle {\sum _{j=1}^{\frac{k}{2}}} \dfrac{ {\tilde{u}}^{(2j-1)}(1)}{(2j-1)!}(|x|-1)^{2j-1}, &{} x \in {\mathbb {B}}^5_2 \setminus {\mathbb {B}}^5, ~k \text { even},\\ 0, &{} x \in {\mathbb {R}}^5 \setminus {\mathbb {B}}^5_2, \end{array}\right. } \end{aligned}$$

and then by means of the cut-off $\chi$ we let

A.2 $$\begin{aligned} Eu:= \chi {\tilde{E}}u. \end{aligned}$$

Note that $E: C^k_{\text {rad}}({\mathbb {B}}^5) \rightarrow C^k_{c,\text {rad}}({\mathbb {R}}^5)$. By denoting with ${\tilde{D}}^iu$ the radial profile of $D^i u$ we have that

A.3 $$\begin{aligned} |{\tilde{u}}^{(j)}(1)| \lesssim \sum _{i=0}^{j} |{\tilde{D}}^iu(1)|. \end{aligned}$$

Furthermore, by the fundamental theorem of calculus,

A.4 $$\begin{aligned} |{\tilde{D}}^{2i+1}u(1)|&\lesssim \left| \int _{0}^{1}\partial _r(r^4 {\tilde{D}}^{2i+1}u(r))\textrm{d}r \right| \lesssim \left( \int _{0}^{1}\left| r^{-4} \partial _r(r^4{\tilde{D}}^{2i+1}u(r)) \right| ^2r^4 \textrm{d}r \right) ^{\frac{1}{2}} \nonumber \\&\lesssim \Vert D^{2i+2}u \Vert _{L^2({\mathbb {B}}^5)}, \end{aligned}$$

and by Hardy’s inequality (see, e.g., [26], Lemma 2.12) we infer that

A.5 $$\begin{aligned} |{\tilde{D}}^{2i}u(1)| \lesssim \Vert D^{2i}u \Vert _{L^2({\mathbb {B}}^5)} + \Vert D^{2i+1}u \Vert _{L^2({\mathbb {B}}^5)}. \end{aligned}$$

Therefore, from (A.3), (A.4) and (A.5), we have that

A.6 $$\begin{aligned} |{\tilde{u}}^{(j)}(1)| \lesssim \sum _{i=1}^{j+1} \Vert D^iu \Vert _{L^2({\mathbb {B}}^5)}. \end{aligned}$$

Now, based on these results, from (A.2) we get that, for $i \leqq k$,

$$\begin{aligned} \Vert D^k Eu \Vert _{L^2({\mathbb {R}}^5 \setminus {\mathbb {B}}^5)} = \Vert D^k Eu \Vert _{L^2({\mathbb {B}}^5_{3/2} \setminus {\mathbb {B}}^5)} \lesssim \sum _{j=0}^{k} \Vert D^iu \Vert _{L^2({\mathbb {B}}^5)}. \end{aligned}$$

Therefore, we finally infer that

$$\begin{aligned} \Vert \partial ^\alpha u \Vert _{L^2({\mathbb {B}}^5)}&\lesssim \Vert \partial ^\alpha Eu \Vert _{L^2({\mathbb {R}}^5)} \lesssim \Vert {\mathcal {F}}[\partial ^\alpha Eu] \Vert _{L^2({\mathbb {R}}^5)} \\ {}&\lesssim \Vert |\cdot |^k Eu \Vert _{L^2({\mathbb {R}}^5)} \lesssim \Vert D^k Eu \Vert _{L^2({\mathbb {R}}^5)} \\ {}&\lesssim \Vert D^k u \Vert _{L^2({\mathbb {B}}^5)} + \Vert D^k u \Vert _{L^2({\mathbb {R}}^5 \setminus {\mathbb {B}}^5)} \lesssim \sum _{j=0}^{k} \Vert D^j u \Vert _{L^2({\mathbb {B}}^5)}, \end{aligned}$$

for all $$u \in C^\infty _{\text {rad}}({\mathbb {B}}^5)$$. $$\square $$

## Appendix B. Equivalence of Sobolev Norms for the Reduced Mass

### Lemma B.1

Let $d \in {\mathbb {N}}$. For every $u={\tilde{u}}(|\cdot |) \in C^\infty _{c,rad }({\mathbb {R}}^d)$ define $w={\tilde{w}}(|\cdot |)$ by

$$\begin{aligned} {\tilde{w}}(r):={r^{-d}} \int _{0}^{r}{{\tilde{u}}}(s)s^{d-1}\textrm{d}s. \end{aligned}$$

Then given $k \in {\mathbb {N}}_0$ we have that

B.1 $$\begin{aligned} \Vert u \Vert _{{\dot{H}}^k({\mathbb {R}}^d)} \simeq \Vert w \Vert _{{\dot{H}}^{k+1}({\mathbb {R}}^{d+2})} \end{aligned}$$

for all $$u \in C^\infty _{c,rad }({\mathbb {R}}^d)$$.

### Proof

The proof relies on the Bessel function representation of the Fourier transform of radial functions. Recall our convention (1.11). Then, for a radial Schwartz function $f={\tilde{f}}(|\cdot |)$, we have that

B.2 $$\begin{aligned} {\mathcal {F}}_df(\xi )=|\xi |^{1-\frac{d}{2}}\int _{0}^{\infty }{\tilde{f}}(r)J_{\frac{d}{2}-1}(r|\xi |)r^{\frac{d}{2}}\textrm{d}r, \end{aligned}$$

(see, e.g., Grafakos [30], p. 429). Now, for $\rho >0$ by partial integration we have

B.3 $$\begin{aligned} \int _{0}^{\infty }{\tilde{u}}(r)J_{\frac{d}{2}-1}(r\rho )r^{\frac{d}{2}}\textrm{d}r&= \int _{0}^{\infty }\big ({\tilde{w}}(r)r^d\big )'J_{\frac{d}{2}-1}(r\rho )r^{1-\frac{d}{2}}\textrm{d}r \nonumber \\&= \rho \int _{0}^{\infty }\!{\tilde{w}}(r)\left( -J'_{\frac{d}{2}-1}(r\rho )\!+\!\frac{\frac{d}{2}-1}{r\rho }J_{\frac{d}{2}-1}(r\rho )\right) r^{\frac{d}{2}+1}\textrm{d}r \nonumber \\&= \rho \int _{0}^{\infty }{\tilde{w}}(r)J_{\frac{d}{2}}(r\rho )r^{\frac{d}{2}+1}\textrm{d}r, \end{aligned}$$

where we used the recurrence relation for *J*-Bessel functions

$$\begin{aligned} J_{\nu +1}(z)=-J'_{\nu }(z)+\frac{\nu }{z}J_{\nu }(z). \end{aligned}$$

According to (B.3) and (B.2) we have that

$$\begin{aligned} \Vert u \Vert _{{\dot{H}}^k({\mathbb {R}}^d)} = \Vert |\cdot |^k {\mathcal {F}}_du \Vert _{L^2({\mathbb {R}}^d)} \simeq \Vert |\cdot |^{k+1} {\mathcal {F}}_{d+2}w \Vert _{L^2({\mathbb {R}}^{d+2})} = \Vert w \Vert _{{\dot{H}}^{k+1}({\mathbb {R}}^{d+2})}. \end{aligned}$$

$$\square $$

## Appendix C. An ODE Result

In this section, we use the following notation

$$\begin{aligned} C_{e}^{\infty }[0,\infty ):= \{ u \in C^{\infty }[0,\infty ): u^{(2k+1)}(0) = 0, k \in {\mathbb {N}}_0 \}, \end{aligned}$$

and note $f \in C_{\textrm{rad }}^{\infty }({\mathbb {R}}^d)$ if and only if $f = {{\tilde{f}}}(|\cdot |)$ with ${{\tilde{f}}} \in C_{e}^{\infty }[0,\infty )$. For $\rho \in [0,\infty )$ we set

$$\begin{aligned} V_0(\rho ):= 2 \rho {{\tilde{\phi }}}(\rho ), \quad V_1(\rho ):= 2\rho {{\tilde{\phi }}}'(\rho ) + 12 {{\tilde{\phi }}}(\rho ), \end{aligned}$$

where ${{\tilde{\phi }}}(\rho ) = \frac{2}{2+\rho ^2}$. Also, we let ${\bar{\omega }}_k$ denote the constant in Equation (4.18).

### Lemma C.1

Let $k \in {\mathbb {N}}$, $k \geqq 3$. Let *f* be an element of $C_{e}^{\infty }[0,\infty )$ with bounded support, and let $\lambda > \max \{2,{\bar{\omega }}_k \}$. Then there exists a function $u \in C^1[0,\infty ) \cap C^{\infty }(0,\infty )$ which solves the equation

C.1 $$\begin{aligned} u''(\rho ) + \left( \frac{4}{\rho } - \frac{1}{2} \rho + V_0(\rho ) \right) u'(\rho ) + \left( V_1(\rho ) - (\lambda + 1) \right) u(\rho ) = f(\rho ) \end{aligned}$$

on the interval $(0,\infty )$, satisfies $u'(0) = 0$, and given $j \in {\mathbb {N}}_0$ obeys the estimate

$$\begin{aligned} |u^{(j)}(\rho )| \lesssim \rho ^{-2-2\lambda - j} \end{aligned}$$

as $$\rho \rightarrow \infty $$.

### Proof

First, we construct a fundamental system for the homogeneous equation

C.2 $$\begin{aligned} u''(\rho ) + \left( \frac{4}{\rho } - \frac{1}{2} \rho + V_0(\rho ) \right) u'(\rho ) + \left( V_1(\rho ) - (\lambda + 1) \right) u(\rho ) = 0. \end{aligned}$$

We note that the origin $\rho = 0$ is a regular singular point. Hence, by the Frobenius method, there is a fundamental system $\{ u_0, u_1 \}$ on $(0,\infty )$, where $u_0$ is analytic at $\rho =0$ with $u_0(0) = 1, u_0'(0)=0$, and $u_1(\rho ) \sim \rho ^{-3}$ near $\rho =0$. To analyze the behavior of solutions at infinity we write the equation in normal form. With $\omega (r):= e^{\frac{r^2}{2}} r^{-2} (1+ 2 r^2)^{-1}$ and $v(r)\omega (r) = u(2r)$, Equation (C.2) transforms into

C.3 $$\begin{aligned} v''(r) - (r^2 + \mu ) v(r) + V(r) v(r) = 0 \end{aligned}$$

with $$\mu = 4\lambda - 5 > 0$$ and

$$\begin{aligned} V(r) = \frac{16}{(1+2r^2)^2} + \frac{8}{1+2r^2} - \frac{2}{r^2}. \end{aligned}$$

By transforming the solutions of Equation (C.2) we obtain a fundamental system $\{v_0,v_1 \}$ for Equation (C.3) with $v_0(r) \sim r^2$ and $v_1(r) \sim r^{-1}$ for $r \rightarrow 0^+$.

For large values of the argument, the situation is more involved. For $r \geqq 1$ and $V =0$, a fundamental system can be given in terms of parabolic cylinder functions $\{ U(\frac{\mu }{2}, \sqrt{2} \cdot ), V(\frac{\mu }{2}, \sqrt{2} \cdot ) \}$, with asymptotic behavior

$$\begin{aligned} U(\tfrac{\mu }{2}, \sqrt{2} r) \sim e^{-\frac{1}{2} r^2} r^{- \frac{1}{2} (\mu +1)}, \quad V(\tfrac{\mu }{2}, \sqrt{2} r) \sim e^{\frac{1}{2} r^2} r^{\frac{1}{2} (\mu -1)}, \end{aligned}$$

for $r \rightarrow \infty$, see for example [47]. Our goal is to construct perturbatively a solution to Equation (C.3), linearly independent of $v_0$, that behaves like $U(\tfrac{\mu }{2}, \sqrt{2} \cdot )$ at infinity. We make this fully explicit by considering a slightly different ‘free’ equation first, namely,

$$\begin{aligned} v''(r) - (r^2 + \mu ) v(r) + Q_{\mu }(r) v(r) = 0, \end{aligned}$$

with potential

$$\begin{aligned} Q_{\mu }(r):= \mu ^{-1} q( \mu ^{-1/2} r), \quad q(r) = \frac{2-3r^2}{4(1+r^2)^2}. \end{aligned}$$

This equation has an explicit fundamental system (see [19], Section 4.1.1),

C.4 $$\begin{aligned} v^{\pm }(r) = \tfrac{1}{\sqrt{2}} \mu ^{-\frac{1}{4}} (1 + \tfrac{r^2}{\mu } )^{-\frac{1}{4}} e^{\pm \mu \xi (\mu ^{-1/2} r)} \end{aligned}$$

with $\xi (r) = \frac{1}{2} \log ( r + \sqrt{1+r^2}) + \frac{1}{2}r \sqrt{1+r^2}$ and Wronskian $W(v^{-},v^{+}) = 1$. Note that

$$\begin{aligned} \mu \xi (\mu ^{-1} r) = \tfrac{r^2}{2} + \tfrac{\mu }{2} \log (r) + c_{\mu } + \varphi _{\mu }(r) \end{aligned}$$

with $c_{\mu } \in {\mathbb {R}}$ and $\varphi _{\mu }(r) = {\mathcal {O}}(r^{-2})$ for $r \rightarrow \infty$, hence

$$\begin{aligned} v^{-}(r) \sim e^{-\frac{1}{2} r^2} r^{- \frac{1}{2} (\mu +1)}, \quad v^{+}(r) \sim e^{\frac{1}{2} r^2} r^{\frac{1}{2} (\mu -1)}, \end{aligned}$$

for $r \rightarrow \infty$. We add $Q_{\mu }$ to both sides of Equation (C.3) and put the potential *V* to the right hand side to obtain

C.5 $$\begin{aligned} v''(r) - (r^2 + \mu ) v(r) + Q_{\mu }(r) v(r) ={\mathcal {O}}(r^{-2}) v(r). \end{aligned}$$

Assuming $$r \geqq 1$$, we show by a perturbative argument the existence of a solution $$v_{\infty }$$ to (C.5) which behaves like $$ v^{-}$$ at infinity. For this, we set up a Volterra iteration by reformulating Equation (C.5) as an integral equation using the variation of constants formula. More precisely, we look for a solution $$v_{\infty }$$ that satisfies

$$\begin{aligned} v_{\infty }(r)&= v^{-}(r) + v^{+}(r) \int _{r}^{\infty } v^{-}(s) {\mathcal {O}}(s^{-2}) v_{\infty }(s) \textrm{d}s \\&\quad - v^{-}(r) \int _{r}^{\infty } v^{+}(s) {\mathcal {O}}(s^{-2}) v_{\infty }(s) \textrm{d}s. \end{aligned}$$

Noting that $v^{-}(r) > 0$ for all $r >0$, we set $h(r):= \frac{ v_{\infty }(r)}{ v^{-}(r)}$ and write the above equation as

C.6 $$\begin{aligned} h(r) = 1 + \int _{r}^{\infty } K(r,s) h(s) \textrm{d}s \end{aligned}$$

where

$$\begin{aligned} K(r,s) :=\left[ \frac{v^{+}(r)}{v^{-}(r)} v^{-}(s)^2 - v^{+}(s) v^{-}(s) \right] {\mathcal {O}}(s^{-2}). \end{aligned}$$

Explicitly,

$$\begin{aligned} K(r,s) = \tfrac{1}{2} \mu ^{-\frac{1}{2}} (1 + \tfrac{s^2}{4})^{-\frac{1}{2}} {\mathcal {O}}(s^{-2}) \left( e^{- 2 \mu (\xi (\mu ^{-\frac{1}{2}} s) - \xi (\mu ^{-\frac{1}{2}} r))} - 1 \right) . \end{aligned}$$

Using the fact that $\xi$ is monotonically increasing, we obtain the bound

$$\begin{aligned} |K(r,s)| \lesssim s^{-3}, \end{aligned}$$

for $$1 \leqq r \leqq s$$. Thus,

$$\begin{aligned} \int _{1}^{\infty } \sup _{r \in [1,s]} |K(r,s)| \textrm{d}s \lesssim 1 \end{aligned}$$

and we can apply standard results on Volterra equations (see, e.g., [50], Lemma 2.4) which yield the existence of a solution *h* on $[1,\infty )$ with $|h(r)| \lesssim 1$ and

$$\begin{aligned} |h(r)-1| \lesssim \int _{r}^{\infty } |K(r,s)| \textrm{d}s \lesssim r^{-2}. \end{aligned}$$

By inspection (see also Remark 4.4 in [18]), one finds that

C.7 $$\begin{aligned} |\partial _r^{k} (h(r)-1)|\lesssim _{k} r^{-2-k} \end{aligned}$$

for all $k \in {\mathbb {N}}$. This yields a smooth solution

C.8 $$\begin{aligned} v_{\infty }(r) = v^{-}(r) [ 1 + {\mathcal {O}}(r^{-2}) ] \end{aligned}$$

to Equation (C.3) on $[1,\infty )$, where the error term behaves like a symbol under differentiation.

Now, by linearity, we have the representation

C.9 $$\begin{aligned} v_{\infty } = c_0 v_0 + c_1 v_1, \end{aligned}$$

for some constants $c_0,c_1 \in {\mathbb {C}}$. Suppose that $c_1 = 0$, i.e., $v_{\infty }$ and $v_0$ are linearly dependent. By transforming back, we would obtain a function $u \in C_{e}^{\infty }[0,\infty )$ with $u(\rho ) = {\mathcal {O}}(\rho ^{-2 - 2\lambda })$ as $\rho \rightarrow \infty$. In particular, $u(|\cdot |)$ would belong to ${\mathcal {C}}$ and satisfy $(\lambda - {\mathcal {L}}_k)u(|\cdot |) = 0$ for some $\lambda > {\bar{\omega }}_k$. This, however, contradicts Equation (4.18) stated in the proof of Proposition 4.7. We conclude that $\{ v_{\infty }, v_0\}$ is a fundamental system for Equation (C.3) on $(0,\infty )$, and we denote by $W:= W(v_{\infty },v_0)(1)$ its Wronskian.

Now we turn to the inhomogeneous Equation (C.1), which transforms into

C.10 $$\begin{aligned} v''(r) - (r^2 + \mu ) v(r) + V(r) v(r) = w(r)^{-1}f(r/2). \end{aligned}$$

By the variation of constants formula we find a particular solution

$$\begin{aligned} v(r)&= \frac{v_0(r)}{W} \int _{r}^{\infty } v_{\infty }(s) e^{-s^2/2} s^2 (1 + 2 s^2) f(\tfrac{s}{2}) \textrm{d}s \\&\quad + \frac{v_{\infty }(r)}{W} \int _0^{r} v_0(s) e^{-s^2/2} s^2 (1 + 2 s^2) f(\tfrac{s}{2}) \textrm{d}s. \end{aligned}$$

Obviously, $v \in C^{\infty }(0,\infty )$. Since *f* has bounded support, the first integral vanishes for large *r* and therefore there is a constant *c* such that $v(r)= cv_{\infty }(r)$ for all large enough *r*. For $r \rightarrow 0$, the first integral converges, hence the behavior of the first term is governed by $v_0$. The second integral is of order ${\mathcal {O}}(r^5)$ which compensates the singular behavior of $v_{\infty }$ at the origin. In particular, there is a constant *C* such that $r^{-2}v(r) \rightarrow C$ and $r^{-1}v'(r) \rightarrow 2C$ when $r \rightarrow 0^{+}$. By transforming back, we obtain a solution $u \in C^1[0,\infty ) \cap C^{\infty }(0,\infty )$. By inspection, $u'(0) = 0$ and $u^{(k)}(\rho ) = {\mathcal {O}}(\rho ^{-2 - 2\lambda - k})$ for $\rho \rightarrow \infty$ and $k \in {\mathbb {N}}_0$. $\square$

## Appendix D. Smoothing of $S(\tau )$ via energy methods

We give an alternative proof of the key estimate (5.11) of Proposition 5.4 for some $\omega _{k+1} > 0$. The estimate (5.10) can be proved similarly. As usual, it is enough to assume $f \in C^\infty _{c,\text {rad}}({\mathbb {R}}^5)$. Denote $\tilde{f}:=(1-{\mathcal {P}}_{X^{k}})f \in {\mathcal {C}} \subset {\mathcal {D}}({\mathcal {L}}_k)$. First, we refine the proof of Theorem 4.1. Namely, for $R\geqq 1$ sufficiently large we have

$$\begin{aligned} \tfrac{1}{2} \tfrac{d}{d\tau } \Vert S(\tau )\tilde{f} \Vert ^2_{X^k}&= {\text {Re}}\langle \partial _{\tau } S(\tau ) \tilde{f}|S(\tau ) \tilde{f} \rangle _{X^k} = {\text {Re}}\langle {\mathcal {L}}_k S(\tau ) \tilde{f}|S(\tau ) \tilde{f}\rangle _{X^k} \\&\leqq - \Vert S(\tau )\tilde{f}\Vert ^2_{\dot{H}^{k+1}} + (-\tfrac{1}{4} + \tfrac{C_k}{R^2}) \Vert S(\tau ) \tilde{f}\Vert ^2_{X^k} + C_{R,k} \sum _{j=0}^{k} \Vert S(\tau )\tilde{f} \Vert ^2_{{\mathcal {G}}((1- {\mathcal {L}})^{j/2})} \\&\leqq - \Vert S(\tau )\tilde{f}\Vert ^2_{\dot{H}^{k+1}} -\tfrac{1}{8} \Vert S(\tau ) \tilde{f}\Vert ^2_{X^k}+ C_{k} e^{-2 \omega _0 \tau } \sum _{j=0}^{k} \Vert \tilde{f} \Vert ^2_{{\mathcal {G}}((1- {\mathcal {L}})^{j/2})} \\&\leqq - \Vert S(\tau )\tilde{f}\Vert ^2_{\dot{H}^{k+1}} -\tfrac{1}{8} \Vert S(\tau ) \tilde{f}\Vert ^2_{X^k} + C_{k} e^{-2 \omega _0 \tau } \Vert \tilde{f} \Vert _{X^k}^2 \\&\leqq - \Vert S(\tau )\tilde{f}\Vert ^2_{\dot{H}^{k+1}} - 2 c \Vert S(\tau ) \tilde{f}\Vert ^2_{X^k} + C_k e^{-4 c \tau } \Vert \tilde{f} \Vert ^2_{X^k}, \end{aligned}$$

for $$c = \frac{1}{2} \min \{\omega _0, \frac{1}{8} \}$$. Hence,

$$\begin{aligned} \tfrac{1}{2} \tfrac{d}{d\tau } \left[ e^{4 c \tau } \Vert S(\tau ) \tilde{f}\Vert ^2_{X^k} \right] \leqq - e^{4c\tau }\Vert S(\tau )\tilde{f}\Vert ^2_{\dot{H}^{k+1}} + C_k \Vert \tilde{f} \Vert ^2_{X^k}, \end{aligned}$$

and integration yields

C.11 $$\begin{aligned} {\begin{matrix} \Vert S(\tau ) \tilde{f}\Vert ^2_{X^k} + 2\int _{0}^{\tau }e^{4cs}\Vert S(s)\tilde{f}\Vert ^2_{\dot{H}^{k+1}}ds &{} \leqq (1 + 2 C_k \tau ) e^{-4 c \tau } \Vert \tilde{f} \Vert ^2_{X^k} \\ &{} \lesssim e^{-2 \omega _k \tau } \Vert \tilde{f} \Vert ^2_{X^k}, \end{matrix}} \end{aligned}$$

for some suitably chosen $\omega _k > 0$ and all $\tau \geqq 0$. Now, by using this estimate we can similarly get

$$\begin{aligned} \tfrac{d}{d\tau } \big ( \tau \Vert S(\tau )\tilde{f} \Vert ^2_{\dot{H}^{k+1}({\mathbb {R}}^5)} \big )&= \Vert S(\tau )\tilde{f} \Vert ^2_{\dot{H}^{k+1}({\mathbb {R}}^5)} + 2\tau {\text {Re}}\langle \partial _{\tau } S(\tau ) \tilde{f}|S(\tau ) \tilde{f} \rangle _{\dot{H}^{k+1}({\mathbb {R}}^5)} \\&= \Vert S(\tau )\tilde{f} \Vert ^2_{\dot{H}^{k+1}({\mathbb {R}}^5)} + 2\tau {\text {Re}}\langle {\mathcal {L}}_k S(\tau ) \tilde{f}|S(\tau ) \tilde{f}\rangle _{\dot{H}^{k+1}({\mathbb {R}}^5)} \\&\leqq C_k(1+\tau ) \Vert S(\tau ) \tilde{f}\Vert ^2_{\dot{H}^{k+1}({\mathbb {R}}^5)} + \tilde{C}_{k} \tau \Vert S(\tau )\tilde{f} \Vert ^2_{X^{k}} \\&\leqq C_k (1+\tau ) \Vert S(\tau ) \tilde{f}\Vert ^2_{\dot{H}^{k+1}({\mathbb {R}}^5)} + \bar{C}_k\tau e^{-2 \omega _{k} \tau } \Vert \tilde{f} \Vert ^2_{X^{k}}. \end{aligned}$$

By integration we get

C.12 $$\begin{aligned} \tau \Vert S(\tau )\tilde{f} \Vert ^2_{\dot{H}^{k+1}({\mathbb {R}}^5)} \lesssim \int _{0}^{\tau }(1+s) \Vert S(s) \tilde{f}\Vert ^2_{\dot{H}^{k+1}({\mathbb {R}}^5)}ds + \tau ^2 \Vert \tilde{f} \Vert ^2_{X^{k}}. \end{aligned}$$

for all $\tau \geqq 0$. Now, to show (5.11) we treat two cases.

**Case 1:** $\tau \in (0,2)$. We get from (C.12) and (C.11) that

$$\begin{aligned} \tau \Vert S(\tau )\tilde{f} \Vert ^2_{\dot{H}^{k+1}({\mathbb {R}}^5)} \lesssim e^{-2 \omega _{k} \tau } \Vert \tilde{f} \Vert ^2_{X^{k}} \end{aligned}$$

for all $$\tau \in (0,2)$$.

**Case 2:** $\tau \geqq 2$. From the semigroup property, the Case 1, and (C.11) we have

$$\begin{aligned} \Vert S(\tau )\tilde{f} \Vert ^2_{\dot{H}^{k+1}({\mathbb {R}}^5)} = \Vert S(1)S(\tau -1)\tilde{f} \Vert ^2_{\dot{H}^{k+1}({\mathbb {R}}^5)} \leqq \Vert S(\tau -1)\tilde{f} \Vert ^2_{X^k} \lesssim e^{-2 \omega _k \tau } \Vert \tilde{f} \Vert ^2_{X^k} \end{aligned}$$

for all $$\tau \geqq 2$$.

From these two cases and (C.11), the estimate (5.11) follows.
